# Supplementary material for: pH-dependence of the Plasmodium falciparum chloroquine resistance transporter is linked to the transport cycle
Source: Nat Commun. 2023 Jul 15;14:4234. doi: 10.1038/s41467-023-39969-2 (PMC10349806; doi:10.1038/s41467-023-39969-2)
Supplement: Supplementary file 1 — supplementary information [file 41467_2023_39969_MOESM1_ESM.pdf]

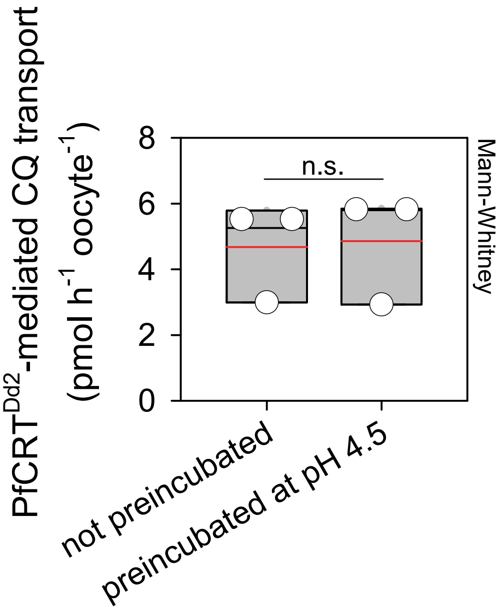

Supplementary Figure 1 I Oocyte viability at pH 4.5. PfCRT^{Dd2}-expressing oocytes were preincubated in transport buffer with a pH of 4.5 before performing the CQ uptake assay in transport buffer with a pH of 6.0 for 1 h. In parallel, water-injected control oocytes were analyzed and the amount of CQ taken up was subtracted from that of the corresponding PfCRT^{Dd2}-expressing oocytes. Results of preincubated oocytes (right) and not preincubated oocytes (left) are compared. A box plot analysis was overlaid over the individual data points, with the median (black line), mean (red lines), and 25% and 75% quartile ranges being shown. The whiskers indicating the 90th and 10th percentile are too small to be resolved on the graph. Each data point represents a biologically independent sample. n.s., not significant according to the two-tailed Mann-Whitney test.

```
1     MKFASKKNNQ KNSSKNDERY RELDNLVQEG NGSRLGGGSC LGKCAHVFKL
51    IFKEIKDNIF IYILSIIYLS VCVIETIFAK RTLNKIGNYS FVTSETHNFI
101   CMIMFFIVYS LFGNKKGNSK ERHRSFNLQF FAISMLDACS VILAFIGLTR
151   TTGNIQSFVL QLSIPINMFF CFLILRYRYH LYNYLGAVII VVTIALVEMK
201   LSFETQEENS IIFNLVLISS LIPVCFSNMT REIVFKKYKI DILRLNAMVS
251   FFQLFTSCLI LPVYTLPFLK ELHLPYNEIW TNIKNGFACL FLGRNTVVEN
301   CGLGMAKLCD DCDGAWKTFA LFSFFSICDN LITSYIIDKF STMTYTIVSC
351   IQGPATAIAY YFKFLAGDVV IEPRLLDFVT LFGYLFGSII YRVGNIILER
401   KKMRNEENED SEGELTNVDS IITQ
```

Protonation/deprotonation | Cation-π interaction | Transmembrane domain

Supplementary Figure 2 I Primary amino acid sequence of PfCRT^{Dd2}. Residues that can undergo pH-induced changes by protonation/deprotonation – Asp, Glu, His – are marked in orange. Aromatic amino acids that may engage in cation-π-interactions (Phe, Tyr, Trp) are marked in green. The transmembrane domains are highlighted in grey.

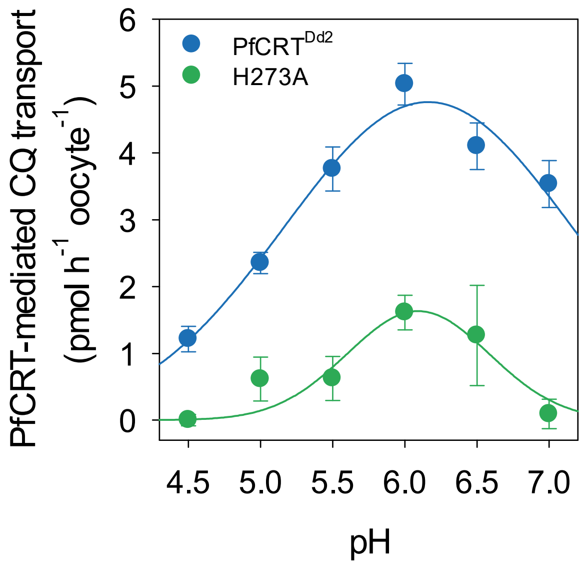

Supplementary Figure 3 I pH-dependency of the H273A mutant compared to PfCRT^{Dd2}. Specific CQ transport activity as a function of the extracellular pH. The extracellular medium contained 50 µM CQ. The means ± SEM of at least three independent biological replicates are shown.

a

```
                                          207              137               329
                                           |                |                 |
Plasmodium falciparum      ..SFETQ E ENSIIF..  ..AISIL D ASSVI..  ..FFNIC D NLITS..
Plasmodium malariae        ..SFETQ E ENSIIF..  ..AISML D ACSVI..  ..FFNIC D NLITS..
Plasmodium ovale curtisi   ..SFETQ E ENSIIF..  ..FISLL D ACSVI..  ..FFNIC D NLITS..
Plasmodium knowlesi        ..SFETQ E ENSIIF..  ..LISLL D ACSVI..  ..FFNIC D NLITS..
Plasmodium vivax           ..SFETQ E ENSIIF..  ..LISLL D ACSVI..  ..FFNIC D NLITS..
Plasmodium gallinaceum     ..SMEIQ E ENSIIF..  ..FISLL D ASTVI..  ..FFNIC D NLVSS..
Plasmodium relictum        ..SMETQ E ENSIIF..  ..LISLL D ASTVI..  ..FFNIC D NLVSS..
Plasmodium blacklocki      ..SFETQ E ENSIIF..  ..AISML D ACSVI..  ..FFNIC D NLITS..
Plasmodium adleri          ..SFETQ E ENSIIF..  ..AISML D ACSVI..  ..FFNIC D NLITS..
Plasmodium gaboni          ..SFETQ E ENSIIF..  ..AISML D ACSVI..  ..FFNIC D NLITS..
Plasmodium billcollinsi    ..SFETQ E ENSIIF..  ..AISML D ACSVI..  ..FFNIC D NLITS..
Plasmodium reichenowi      ..SFETQ E ENSIIF..  ..AISLL D ACSVI..  ..FFNIC D NLITS..
Plasmodium inui            ..SFETQ E ENSIVF..  ..LISML D ACSVI..  ..FFNIC D NLITS..
Plasmodium fragile         ..SFETQ E ENSIVF..  ..AISML D ACSVI..  ..FFNIC D NLITS..
Plasmodium coatneyi        ..SFETQ E ENSIVF..  ..LISLL D ACSVI..  ..FFNIC D NLITS..
Plasmodium cynomolgi       ..SFETQ E ENSIVF..  ..LISLL D ACSVI..  ..FFNIC D NLITS..
Plasmodium praefalciparum  ..SFETQ E ENSIIF..  ..AISML D ACSVI..  ..FFNIC D NLITS..
Plasmodium brasilianum     ..SFETQ E ENSIIF..  ..AISIL D ASSVI..  ..FFNIC D NLITS..
Plasmodium berghei         ..SFETQ G ENSIIF..  ..LISLL D ASTVI..  ..FFNIC D NLLAC..
Plasmodium yoelii yoelii   ..SFETQ S ENSIIF..  ..LISLL D ASTVI..  ..FFNIC D NLLAC..
Plasmodium chabaudi        ..SYETQ S DNSIIF..  ..LISLL D ASTVI..  ..FFNIC D NLLVC..
Plasmodium vinckei vinckei ..SYETQ S DNSIIF..  ..LISLL D AATVI..  ..FFNIC D NLLAC..
```

b

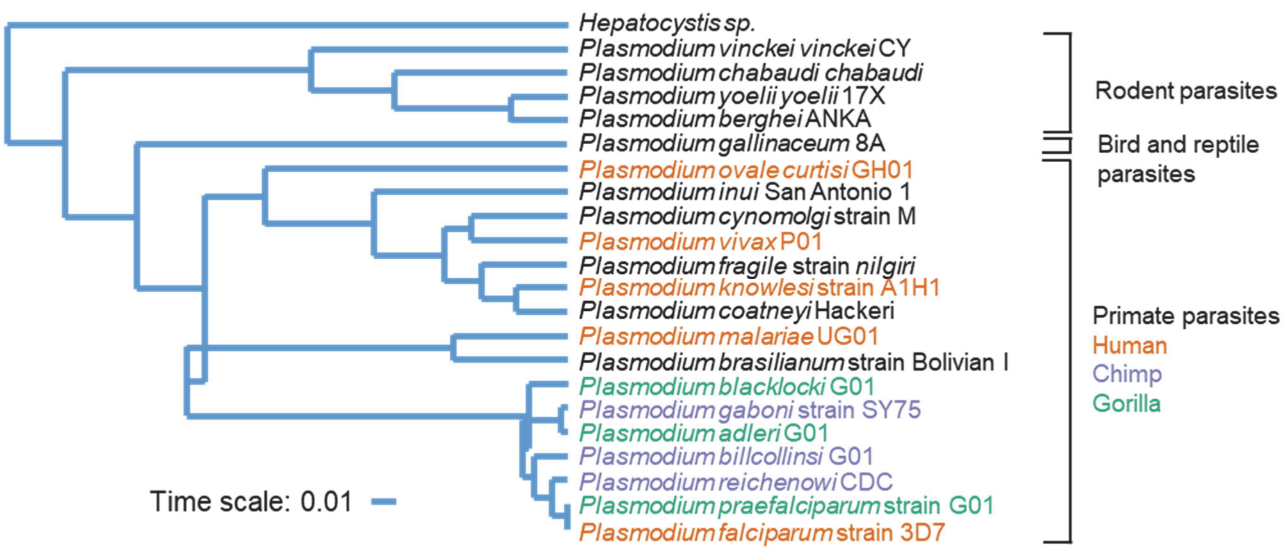

Supplementary Figure 4 I Conservation of E207, D137 and D329 among CRT of Plasmodium species. a, Sequence alignment of CRT from different Plasmodium species. E207, D137 and D329 are highlighted. **b**, bootstrap analysis showing the evolutionary relationship of the CRT orthologs. Data were obtained from plasmodb.org (April 2023).

```
3D7   MKFASKKNNQKNSSKNDERYRELDNLVQEGNGSRLGGGSCLGKCAHVFKLIFKEIKDNIF   60
Dd2   MKFASKKNNQKNSSKNDERYRELDNLVQEGNGSRLGGGSCLGKCAHVFKLIFKEIKDNIF   60
7G8   MKFASKKNNQKNSSKNDERYRELDNLVQEGNGSRLGGGSCLGKCAHVFKLIFKEIKDNIF   60
      ************************************************************

3D7   IYILSIIYLSVCVMNKIFAKRTLNKIGNYSFVTSETHNFICMIMFFIVYSLFGNKKGNSK   120
Dd2   IYILSIIYLSVCVIETIFAKRTLNKIGNYSFVTSETHNFICMIMFFIVYSLFGNKKGNSK   120
7G8   IYILSIIYLSVSVMNTIFAKRTLNKIGNYSFVTSETHNFICMIMFFIVYSLFGNKKGNSK   120
      ***********.*::.********************************************

3D7   ERHRSFNLQFFAISMLDACSVILAFIGLTRTTGNIQSFVLQLSIPINMFFCFLILRYRYH   180
Dd2   ERHRSFNLQFFAISMLDACSVILAFIGLTRTTGNIQSFVLQLSIPINMFFCFLILRYRYH   180
7G8   ERHRSFNLQFFAISMLDACSVILAFIGLTRTTGNIQSFVLQLSIPINMFFCFLILRYRYH   180
      ************************************************************

3D7   LYNYLGAVIIVVTIALVEMKLSFETQ E ENSIIFNLVLISALIPVCFSNMTREIVFKKY   238
Dd2   LYNYLGAVIIVVTIALVEMKLSFETQ E ENSIIFNLVLISSLIPVCFSNMTREIVFKKY   238
7G8   LYNYLGAVIIVVTIALVEMKLSFETQ E ENSIIFNLVLISSLIPVCFSNMTREIVFKKY   238
      ************************** * ************:******************

3D7   KIDILRLNAMVSFFQLFTSCLILPVYTLPFLKQLHLPYNEIWTNIKNGFACLFLGRNTVV   298
Dd2   KIDILRLNAMVSFFQLFTSCLILPVYTLPFLKELHLPYNEIWTNIKNGFACLFLGRNTVV   298
7G8   KIDILRLNAMVSFFQLFTSCLILPVYTLPFLKQLHLPYNEIWTNIKNGFACLFLGRNTVV   298
      ********************************:***************************

3D7   ENCGLGMAKLCDDCDGAWKTFALFSFFNICDNLITSYIIDKFSTMTYTIVSCIQGPAIAI   358
Dd2   ENCGLGMAKLCDDCDGAWKTFALFSFFSICDNLITSYIIDKFSTMTYTIVSCIQGPATAI   358
7G8   ENCGLGMAKLCDDCDGAWKTFALFSFFDICDNLITSYIIDKFSTMTYTIVSCIQGPALAI   358
      ***************************.***************************** **

3D7   AYYFKFLAGDVVREPRLLDFVTLFGYLFGSIIYRVGNIILERKKMRNEENEDSEGELTNV   418
Dd2   AYYFKFLAGDVVIEPRLLDFVTLFGYLFGSIIYRVGNIILERKKMRNEENEDSEGELTNV   418
7G8   AYYFKFLAGDVVREPRLLDFVTLFGYLFGSIIYRVGNIILERKKMRNEENEDSEGELTNV   418
      ************ ***********************************************

3D7   DSIITQ   424
Dd2   DSIITQ   424
7G8   DSIITQ   424
      ******
```

Supplementary Figure 5 I Alignment of the PfCRT variants from the wild type *P. falciparum* line 3D7 and the chloroquine resistant lines Dd2 and 7G8.

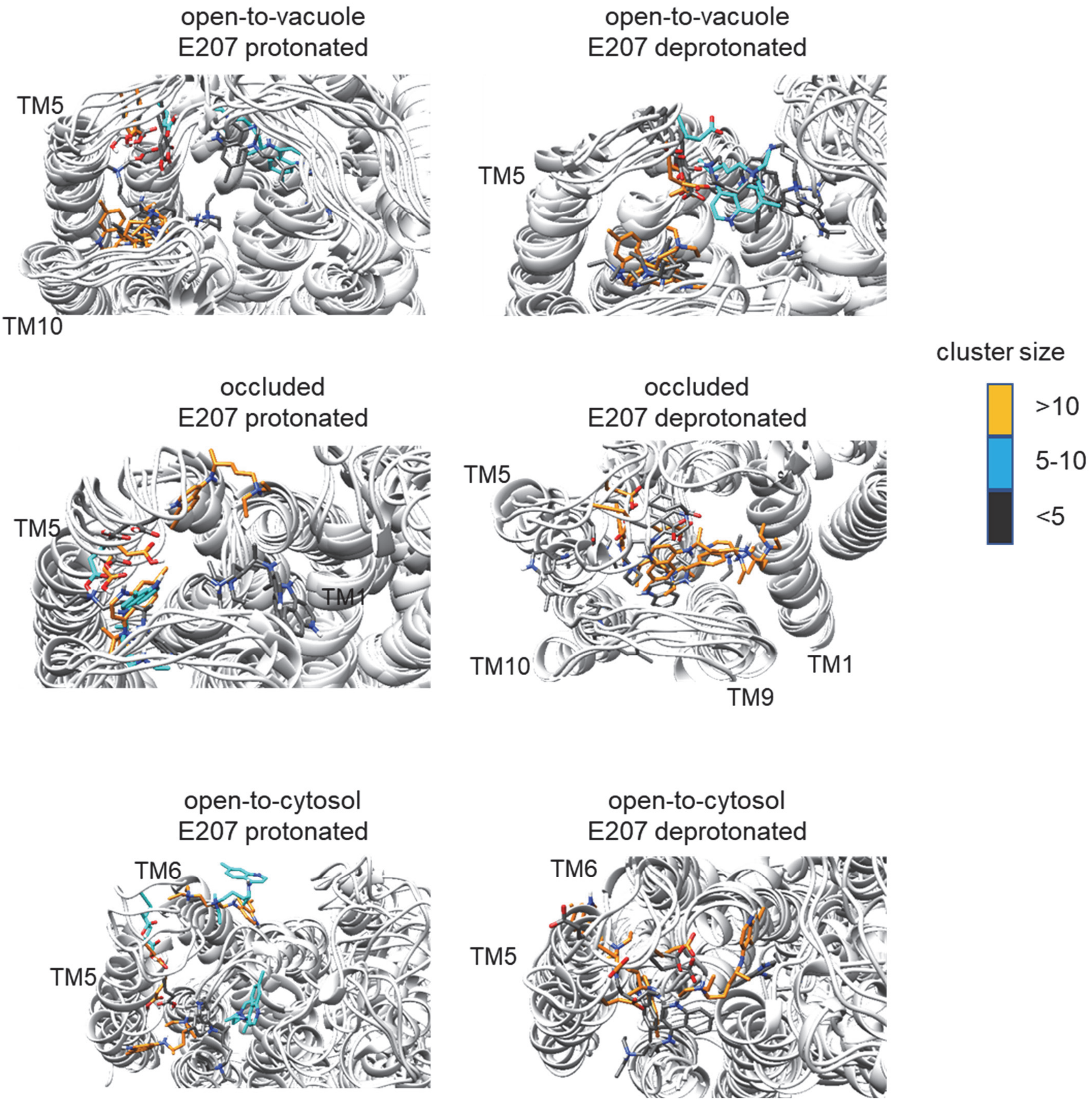

Supplementary Figure 6 I Predicted binding modes of CQ for different conformations of PfCRT (open-to-vacuole, occluded, open-to-cytosol) and different protonation states of E207. Protein configurations were obtained from three independent 200-ns MD simulations, and clustered. CQ was docked to the centers of the clusters. CQ is colored according to the size of the cluster to which it was docked. Docking was performed using AutoDock Vina.

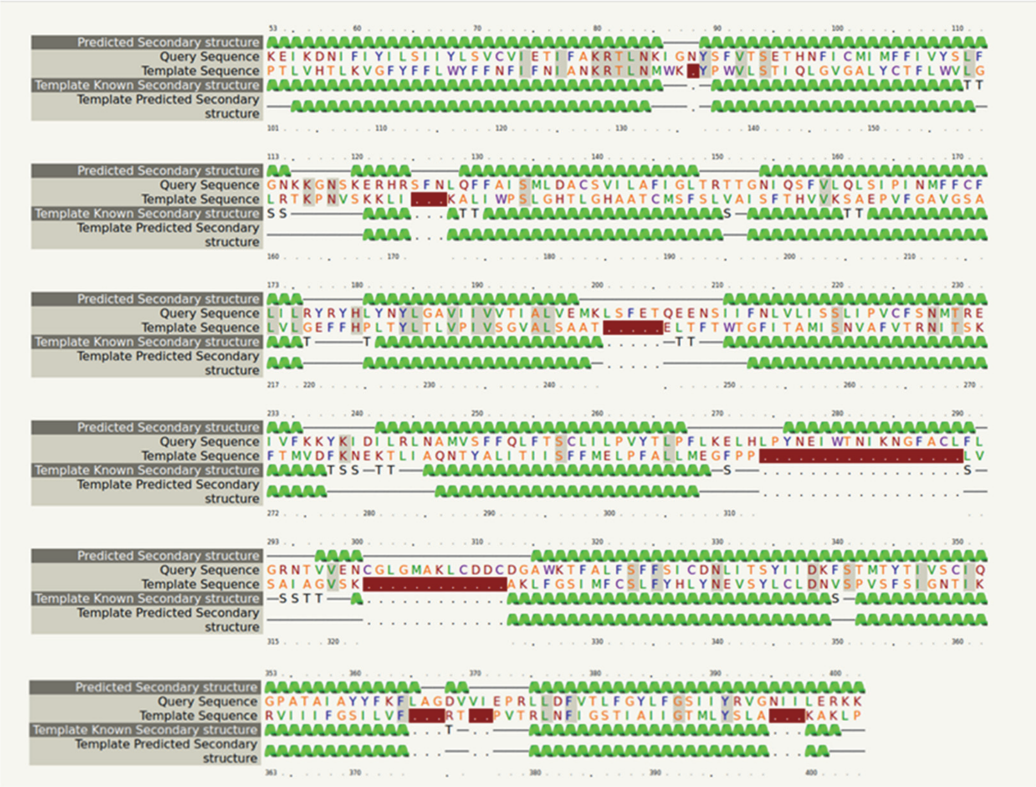

Supplementary Figure 7 I. Sequence alignment between triose-phosphate/phosphate translocator (PDB ID 5Y79, template sequence) and PfCRT^{Dd2} (query sequence). Positions with identical residues are shaded in gray. The sequence identity between PfCRT^{Dd2} and the protein in PDB 5Y79 is 15%.

a

open-to-vacuole conformation of PfCRT, with E207 protonated

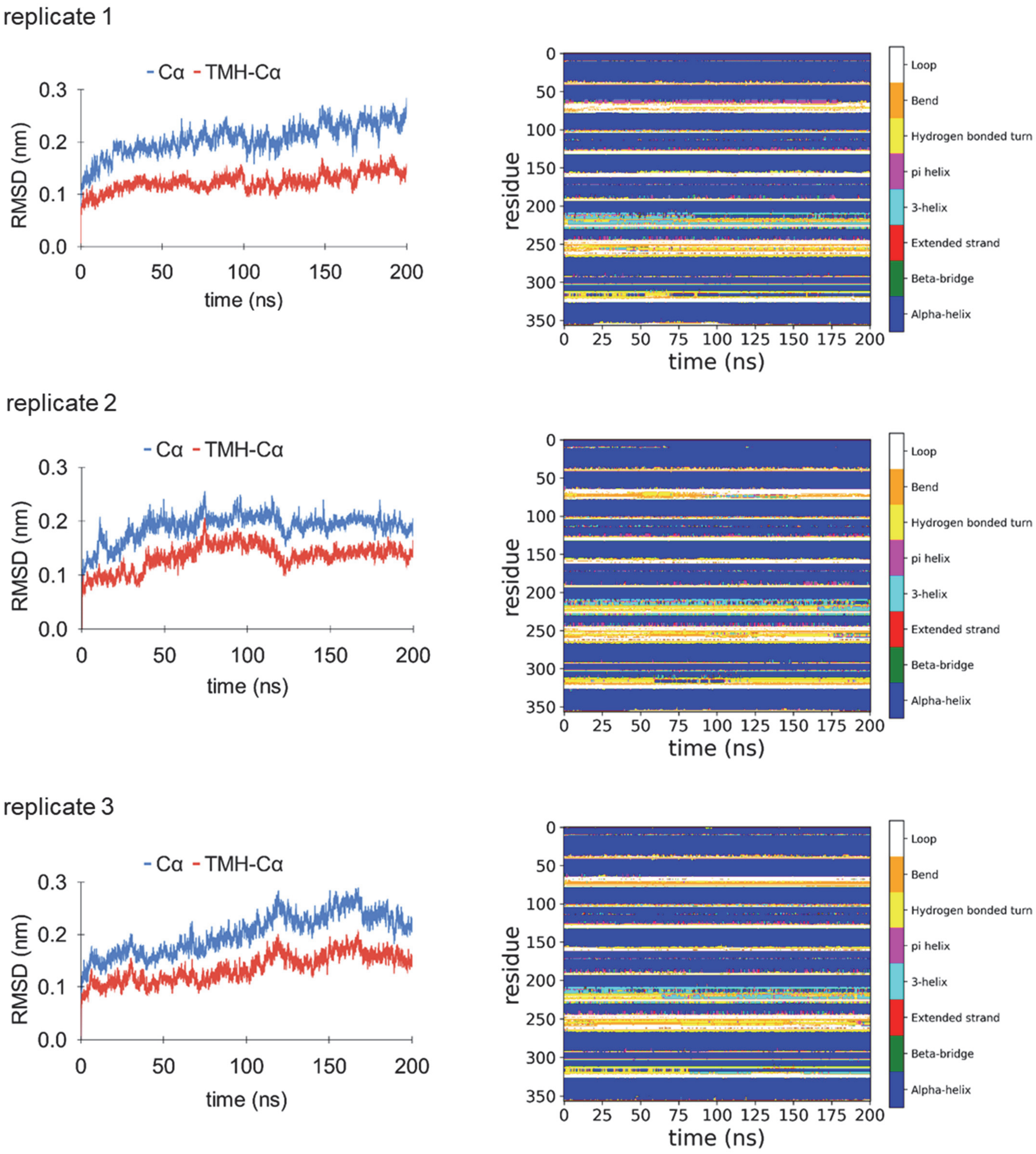

b open-to-vacuole conformation of PfCRT, with E207 deprotonated

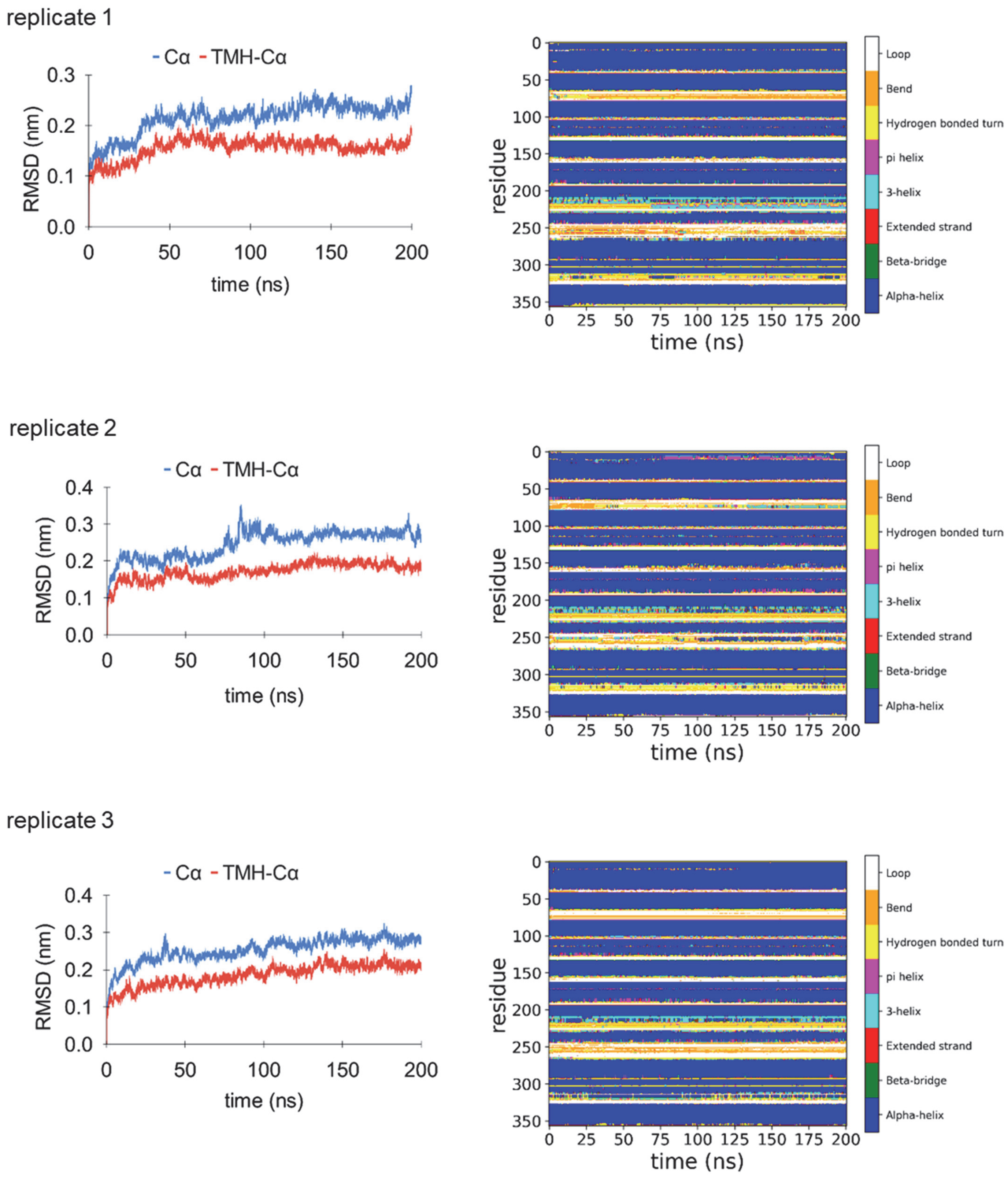

C

occluded conformation of PfCRT, with E207 protonated

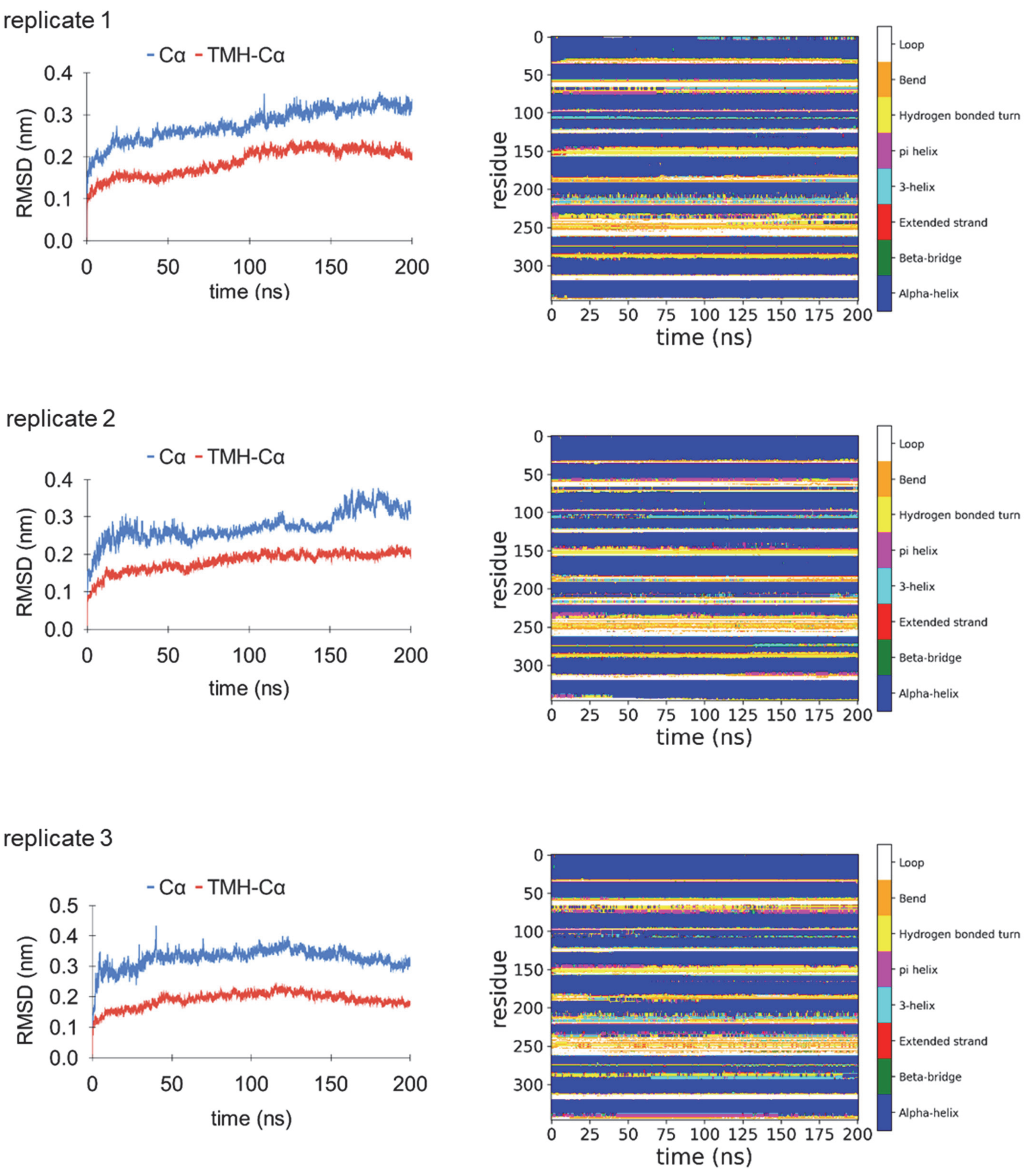

d

occluded conformation of PfCRT, with E207 deprotonated

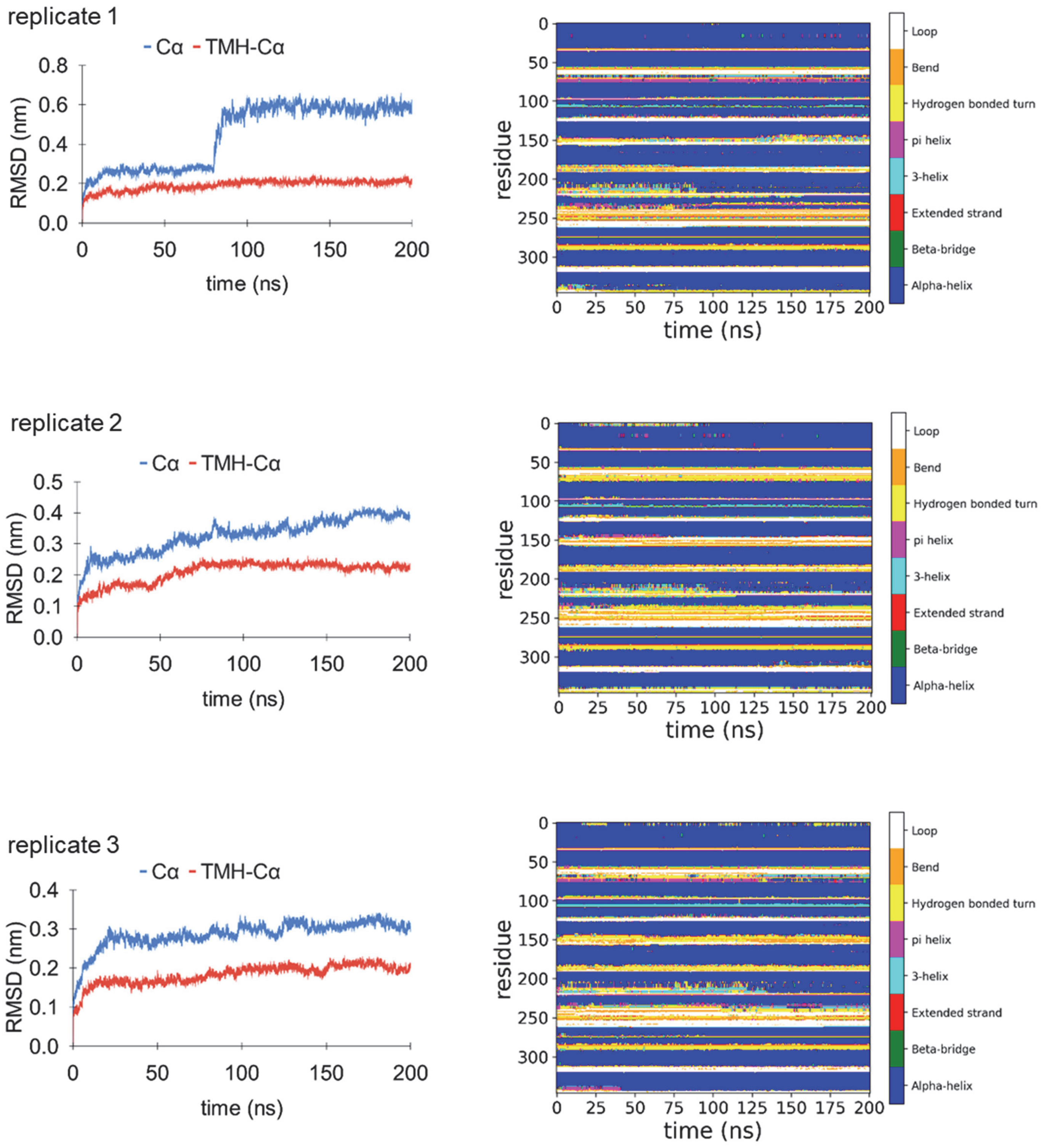

e

open-to-cytoplasm conformation of PfCRT, with E207 protonated

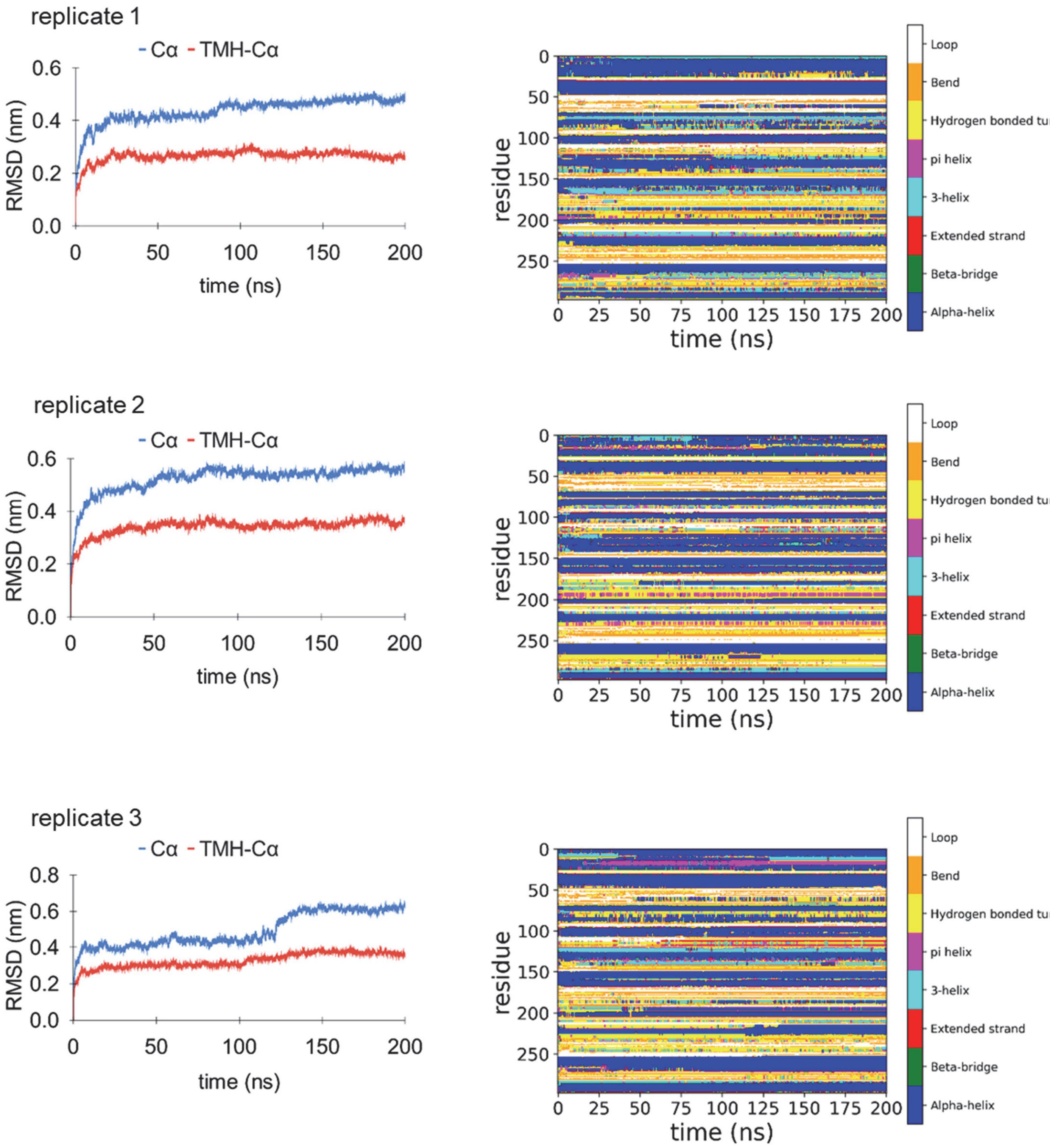

f

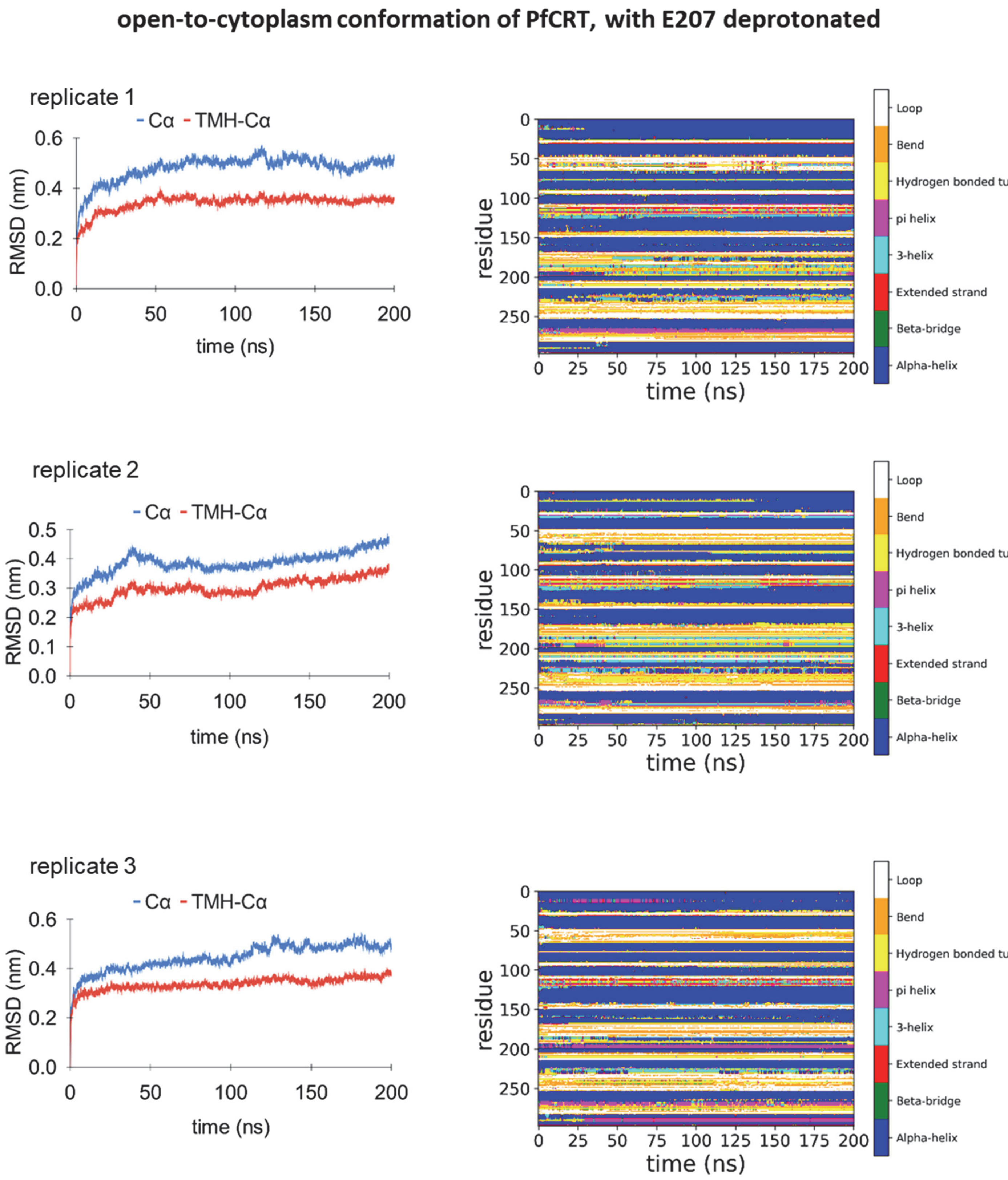

Supplementary Figure 8 I Quality assessment of protein structure in MD simulations of the open-to-vacuole, occluded, and open-to-cytoplasm conformation of PfCRT^{Dd2}, with

E207 being deprotonated or protonated. **a**, open-to-vacuole conformation, with E207 protonated; **b**, open-to-vacuole conformation, with E207 deprotonated; **c**, occluded conformation, with E207 protonated; **d**, occluded conformation, with E207 deprotonated; **e**, open-to-cytoplasm conformation, with E207 protonated; **f**, open-to-cytoplasm conformation, with E207 deprotonated; (left) Root mean squared deviation (RMSD) of alpha-carbon atoms (Cα) of the whole protein, and of transmembrane helices. (right) DSSP analysis, showing the secondary structure of each residue throughout the MD simulation. Three independent replicates are shown for each condition. The change in RMSD observed in one replicate of the occluded conformation, with E207 deprotonated, is due to a conformational change around residue 300, which is a disordered region in the protein (see also Supplementary Figure 19).

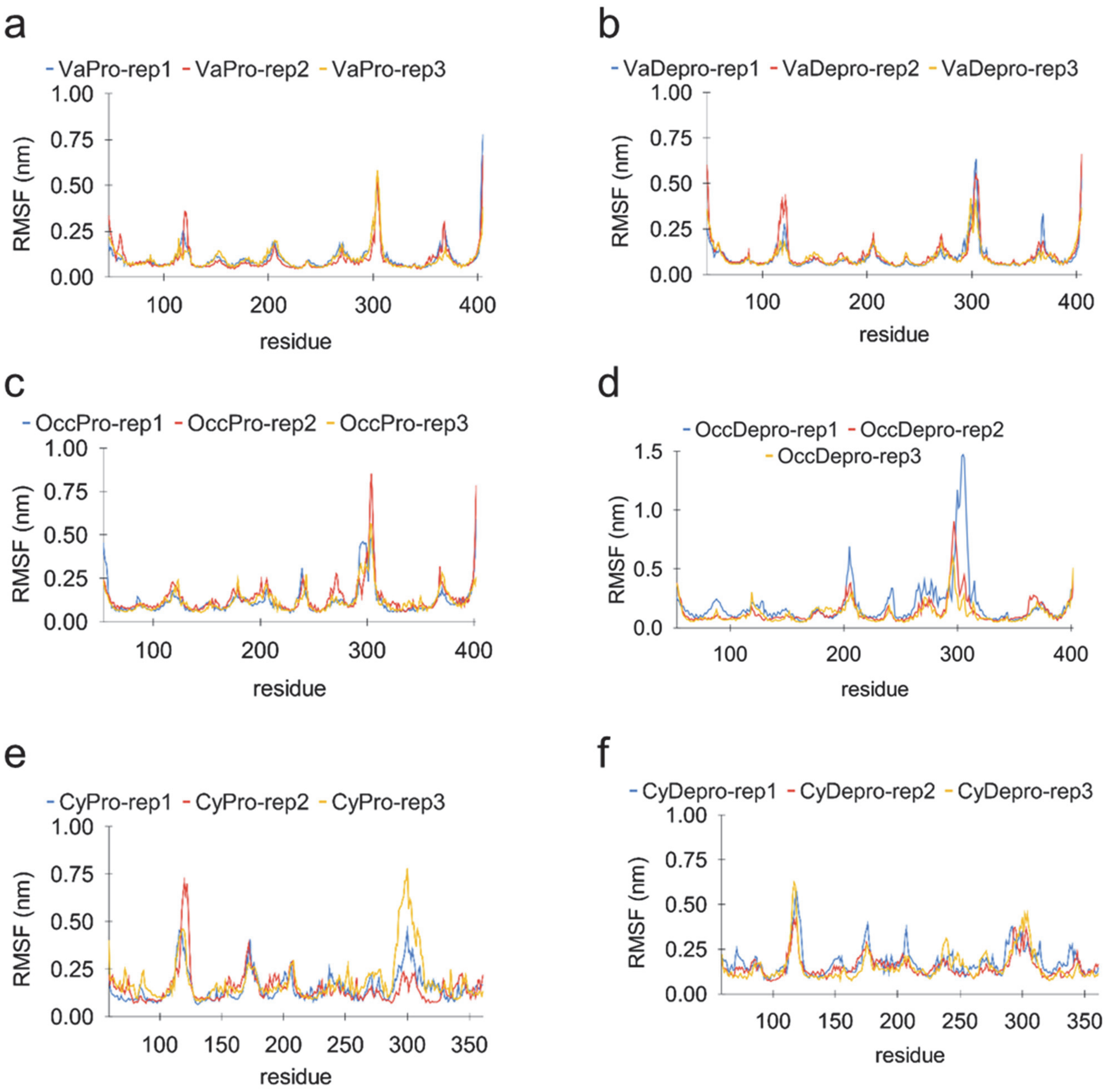

Supplementary Figure 9 l Root mean square fluctuation (RMSF) of the alpha carbons of each residue in 200-ns MD simulations of the three conformations of PfCRT. Each color represents an independent MD simulation. **a**, open-to-vacuole, E207 protonated, **b**, open-to-vacuole, E207 deprotonated, **c**, occluded, E207 protonated, **d**, occluded, E207 deprotonated, **e**, open-to-cytoplasm, E207 protonated, **f**, open-to-cytoplasm, E207 deprotonated. All the transmembrane helices were stable, and the fluctuation were mostly in the random coils connecting the helices.

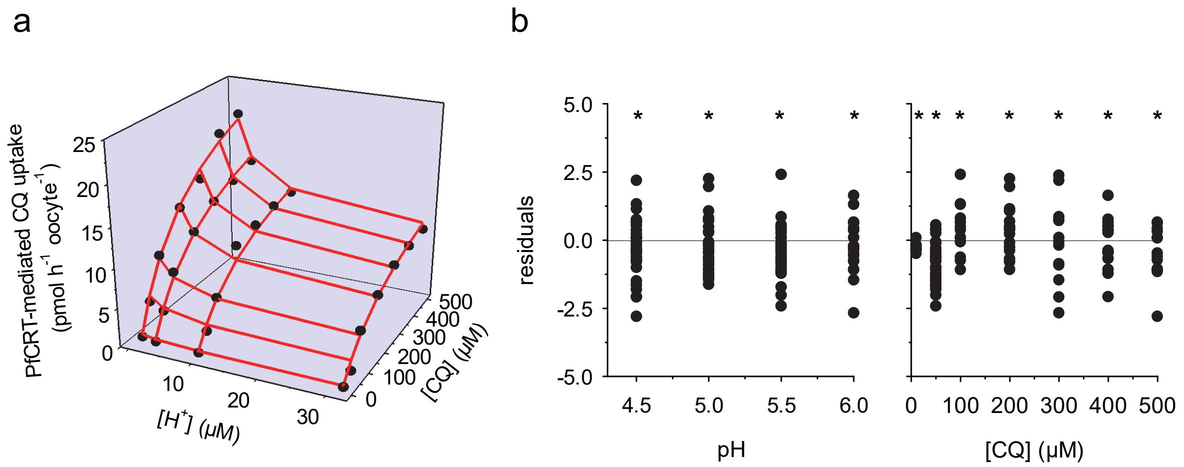

Supplementary Figure 10 I Modeling the interaction between PfCRT^{Dd2}, CQ, and protons using the partial noncompetitive inhibition equation. a, To test how well the partial noncompetitive inhibition model fit the kinetic data presented in Fig. 4a, the corresponding equation was solved using the kinetic parameters presented in Table 1. The resulting predictions (*red lines*) were then displayed as a three-dimensional plot, with the experimentally derived values (black circles) shown for comparison. **b**, The difference between the experimentally derived and the predicted values was calculated, and the resulting residuals are displayed as a function of the proton concentration (pH) and the CQ concentration. Normal distribution of the residuals was tested using the Shapiro-Wilk Normality test. *; normality passed.

a

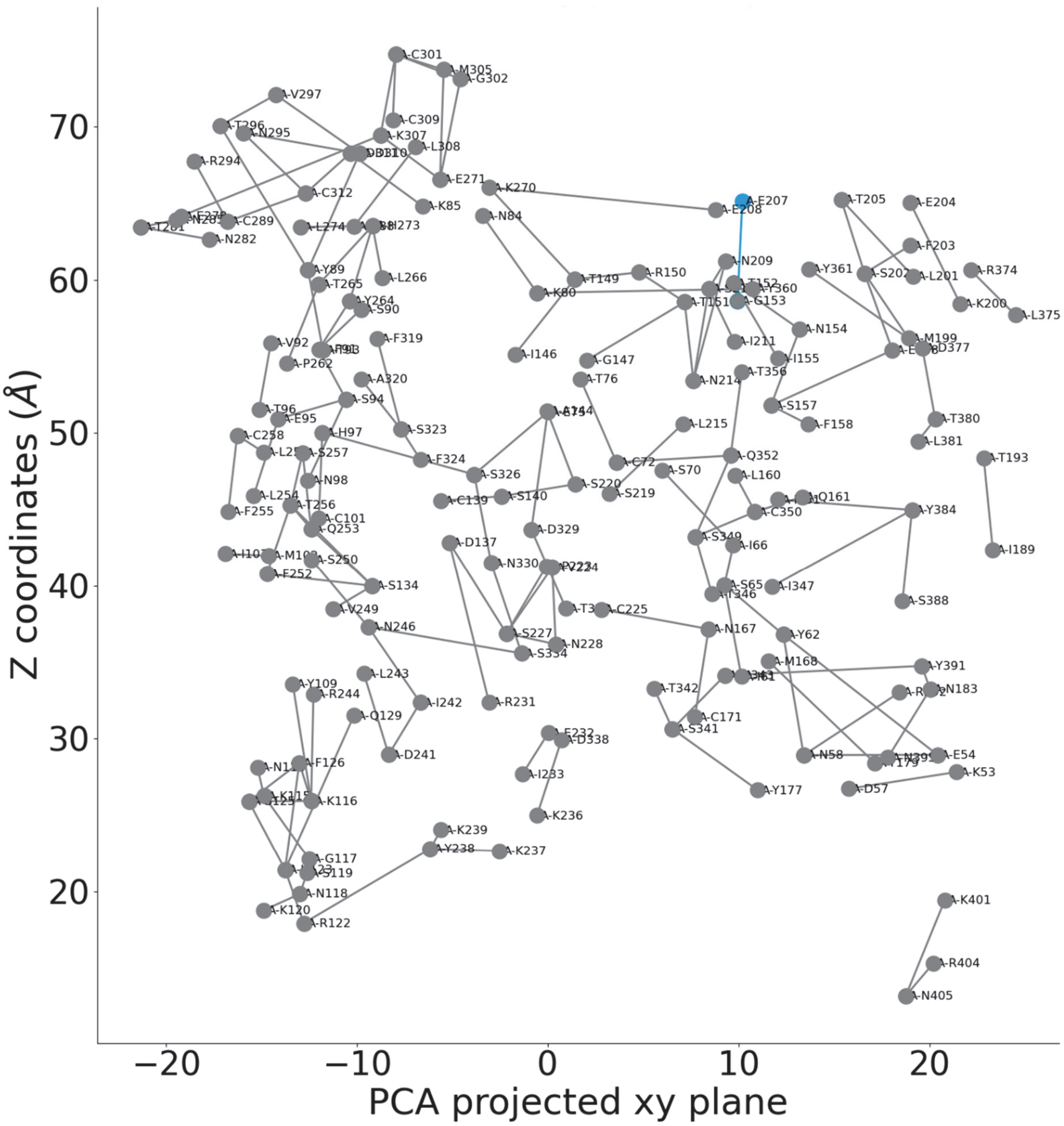
Z coordinates (Å)
70
60
50
40
30
20
−20
−10
0
10
20
PCA projected xy plane

b

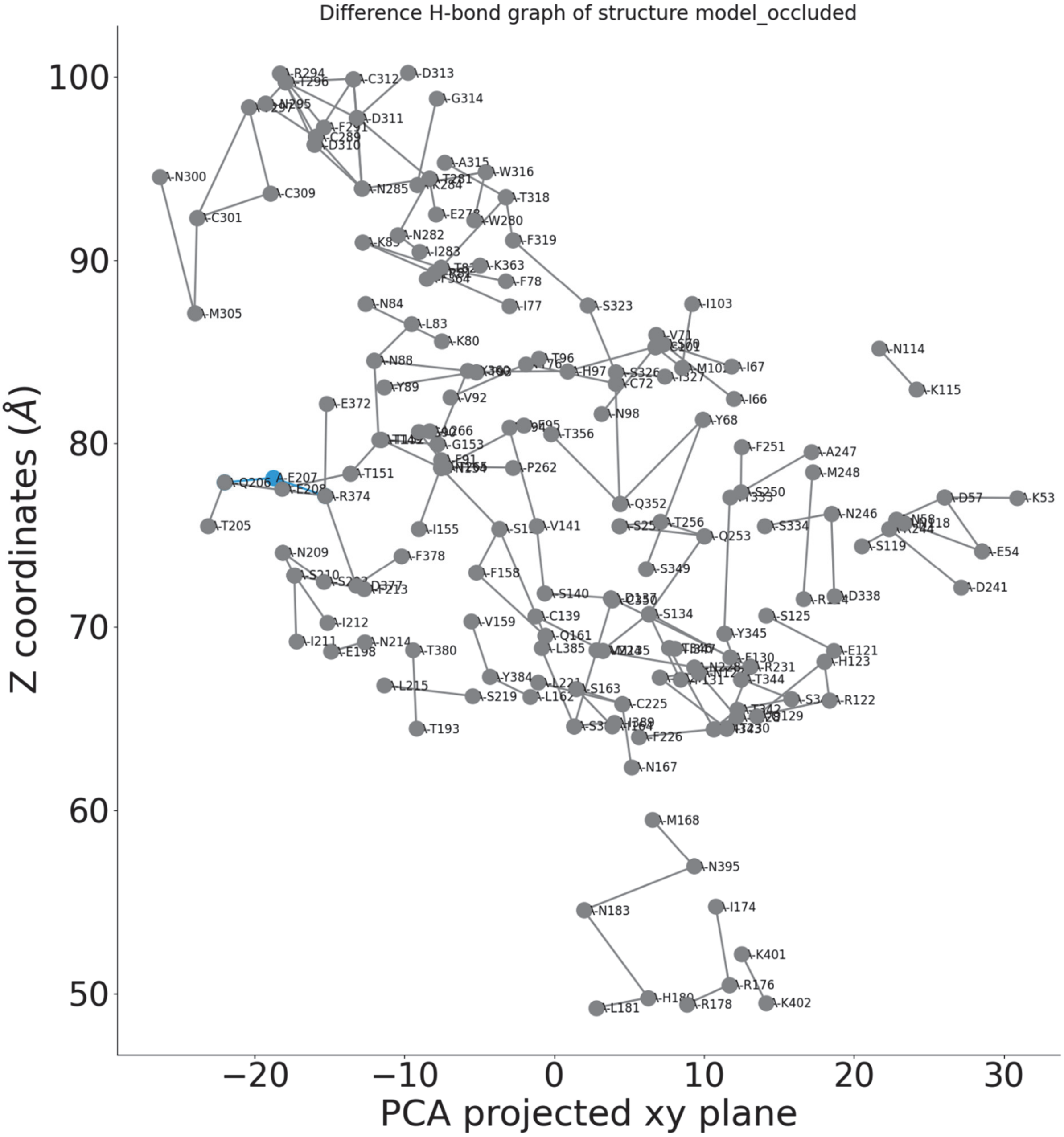

Difference H-bond graph of structure model_occluded
Z coordinates (Å)
PCA projected xy plane
100
90
80
70
60
50
−20
−10
0
10
20
30

c

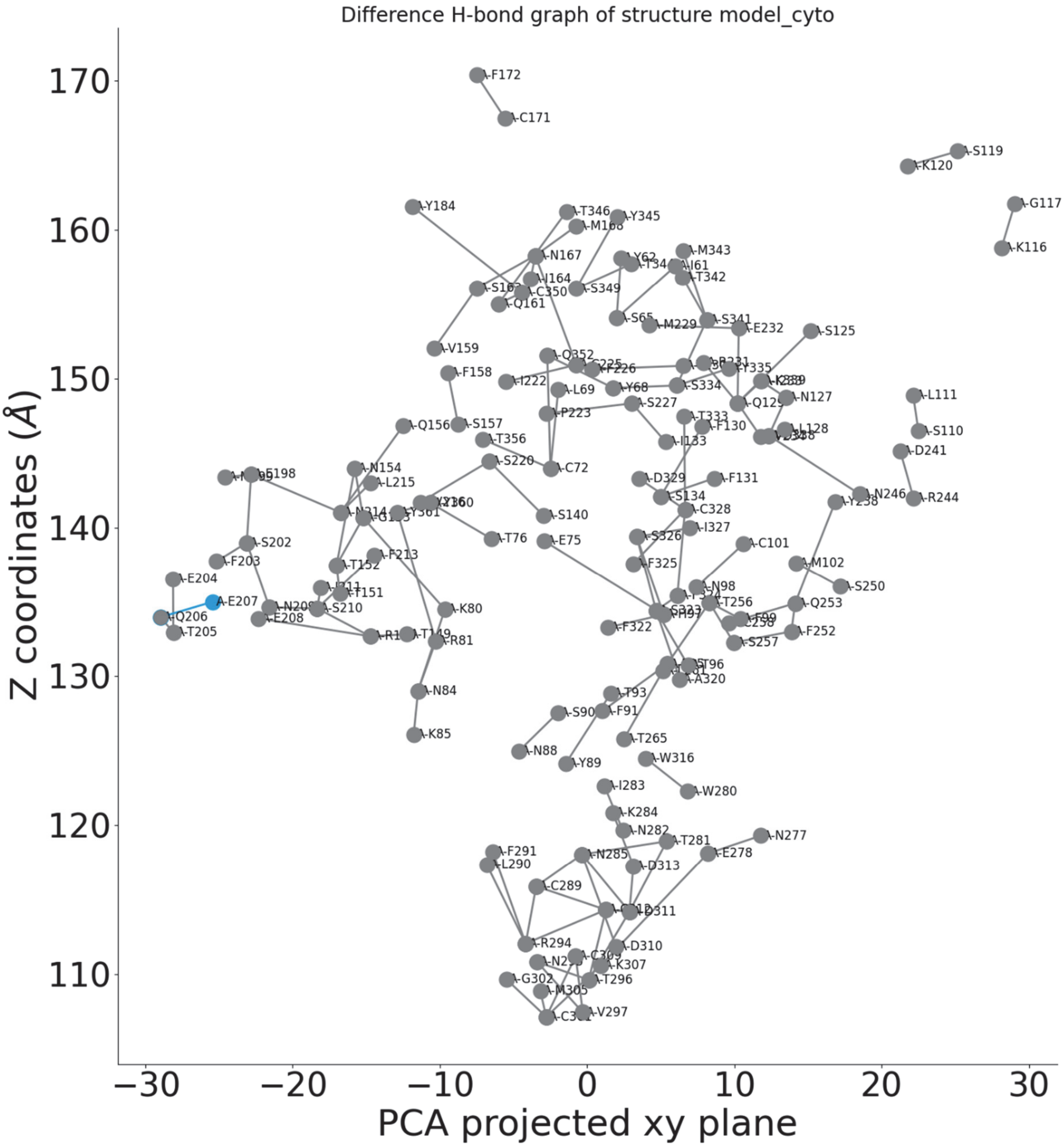

Supplementary Figure 11 I Graphical representation of all the hydrogen bond networks identified in PfCRT^{Dd2}. **a** open-to-vacuole model, **b** occluded state model, **c** open-to-cytoplasm model. Each node represents a residue, and lines represent the hydrogen bonds between residues. Hydrogen bonds involving E207 are highlighted in blue.

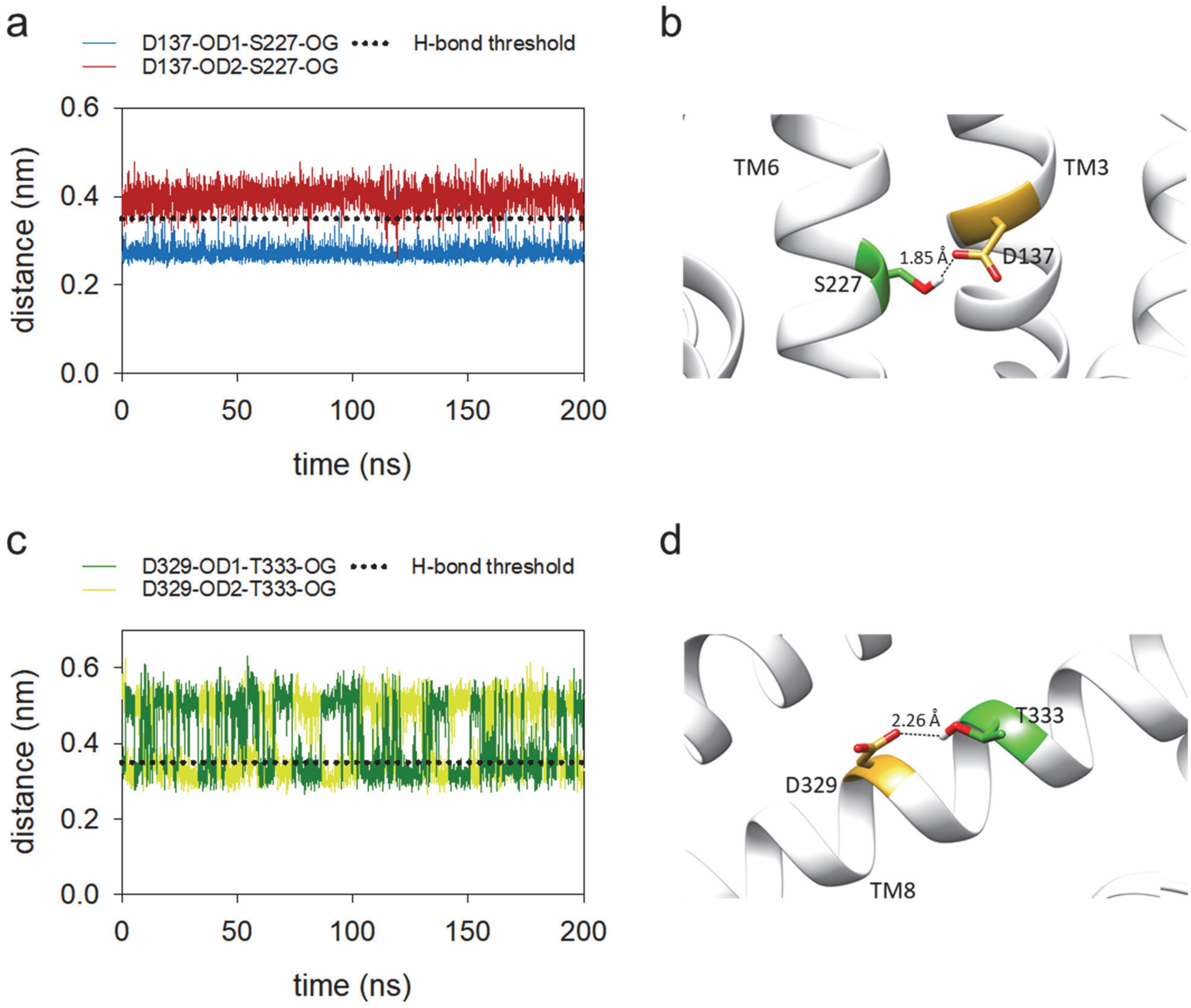

Supplementary Figure 12 I Role of D137 and D329 in proton transfer function of PfCRT
a, Distance between hydrogen bond donors and acceptors of D137-OD1/OD2 and S227-OG, in one MD simulation of the open-to-vacuole conformation of PfCRT, with E207 deprotonated. Black traced lines indicate the threshold distance for the formation of a hydrogen bond (0.35 nm). **b**, Hydrogen bond between D137 and S227 in one MD simulation of the open-to-vacuole conformation of PfCRT, with E207 deprotonated. **c** Distance between hydrogen bond donors and acceptors of D329-OD1/OD2 and T333-OG, in one MD simulation of the open-to-vacuole conformation of PfCRT, with E207 deprotonated. Black traced lines indicate the threshold distance for the formation of a hydrogen bond (0.35 nm).**d**, Hydrogen bond between D329 and T333 in one MD simulation of the open-to-vacuole conformation of PfCRT, with E207 deprotonated.

open-to-vacuole conformation

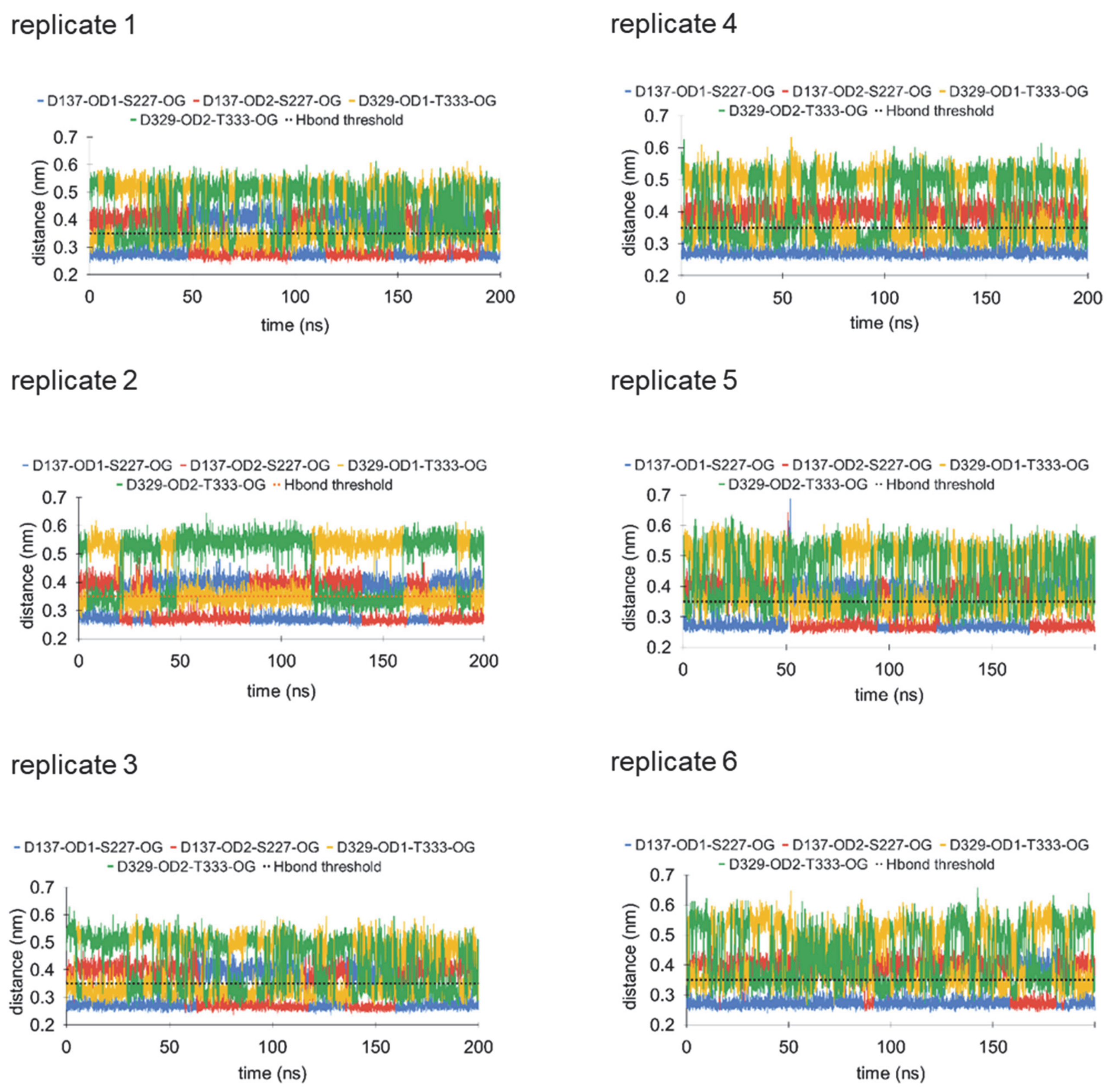

occluded conformation

replicate 1

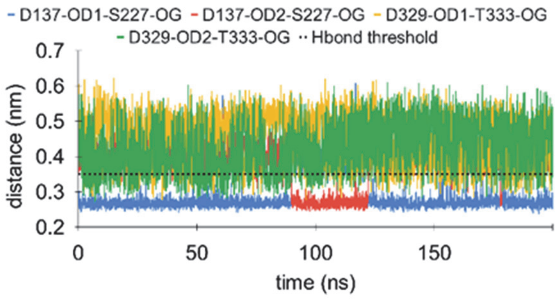

replicate 4

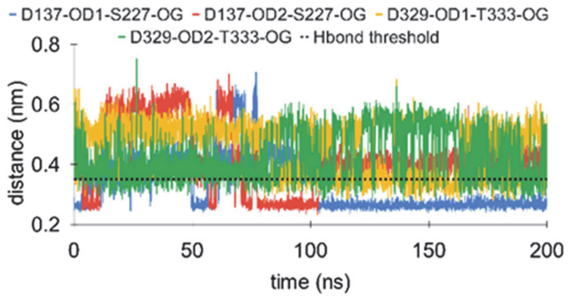

replicate 2

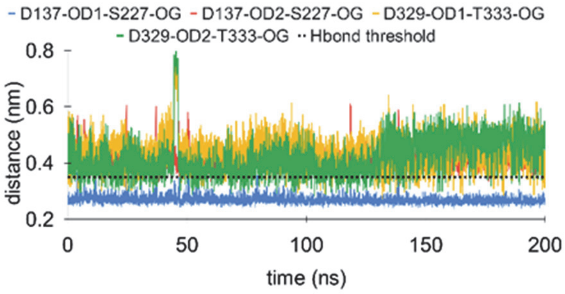

replicate 5

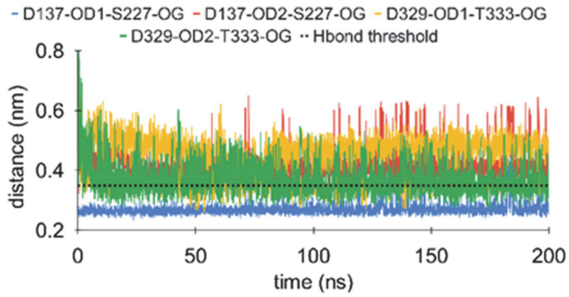

replicate 3

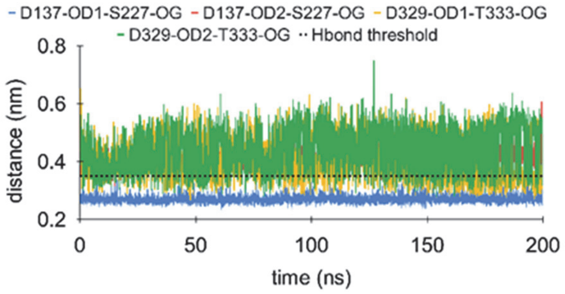

replicate 6

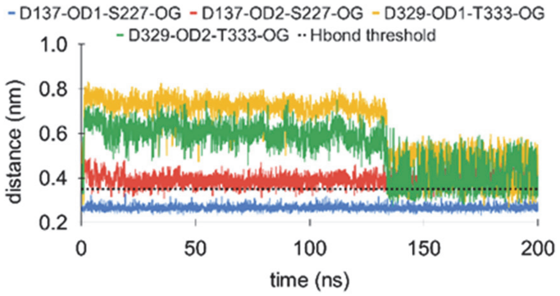

occluded conformation

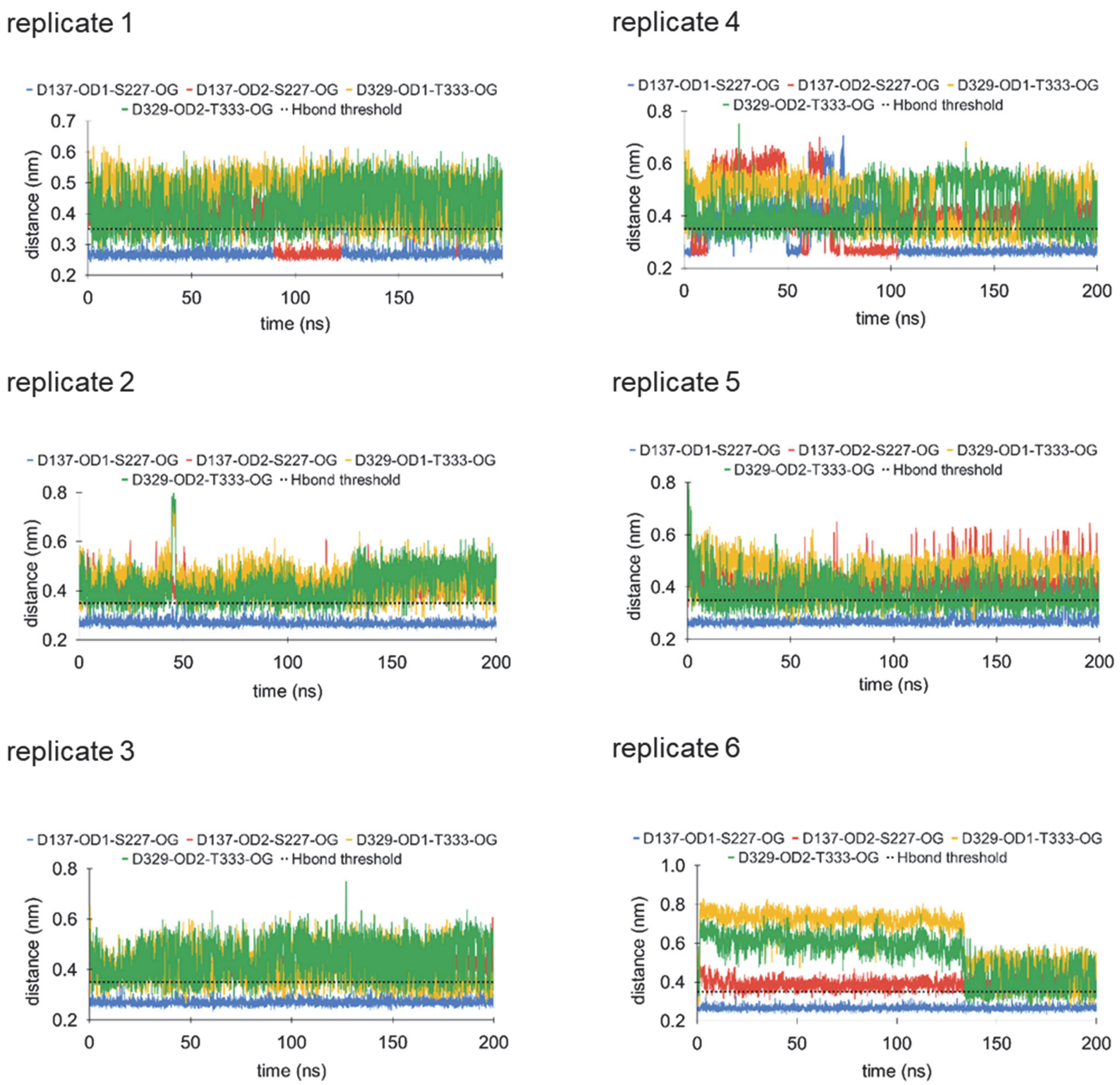

Supplementary Figure 13 I Distance between hydrogen bond donors and acceptors, involving D137, D329 and S227, T333, respectively, in MD simulations of different conformations of PfCRT^{Dd2}. Replicates 1 to 3 refer to models with E207 protonated and replicates 4 to 6 refer to models with E207 deprotonated. Dotted lines indicate the threshold distance for the formation of a hydrogen bond (0.35 nm).

open-to-vacuole conformation

E207 protonated

replicate 1

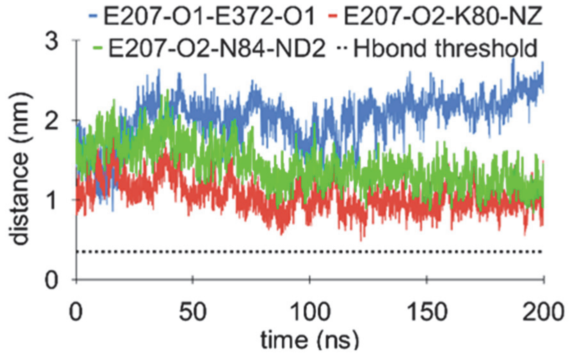

replicate 2

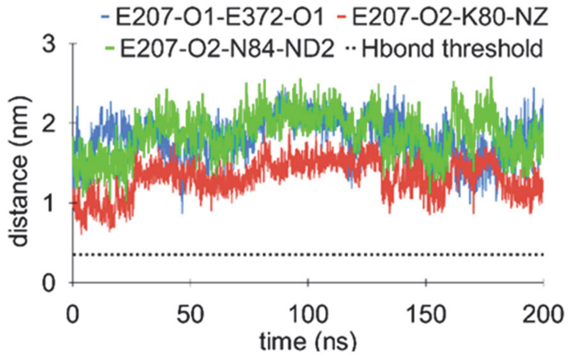

replicate 3

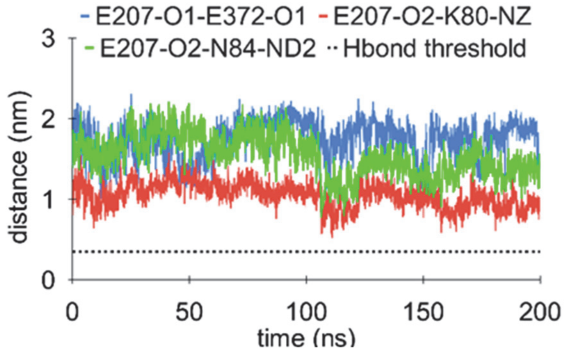

E207 deprotonated

replicate 1

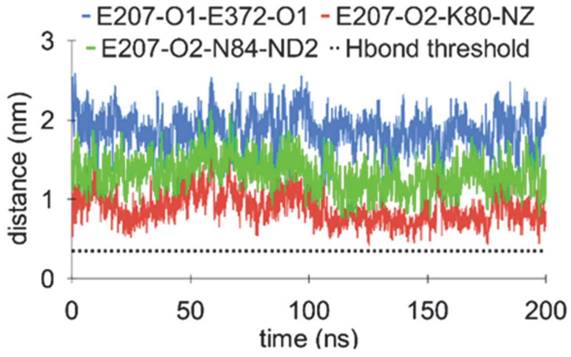

replicate 2

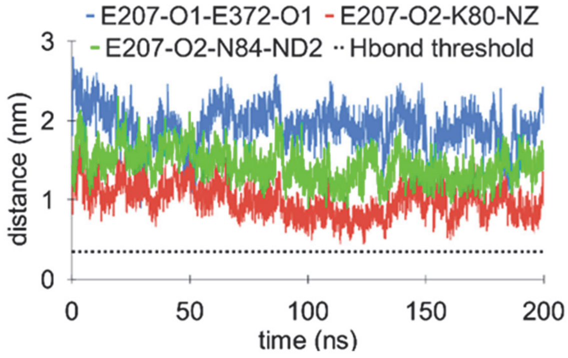

replicate 3

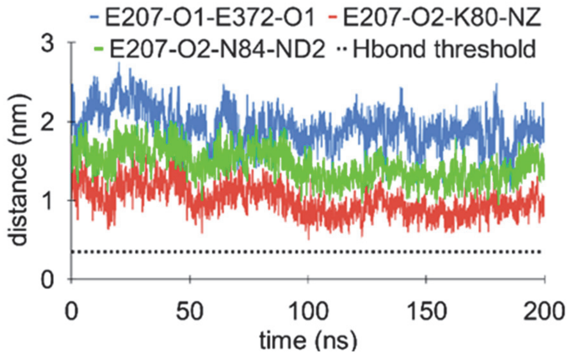

occluded conformation

E207 protonated

E207 deprotonated

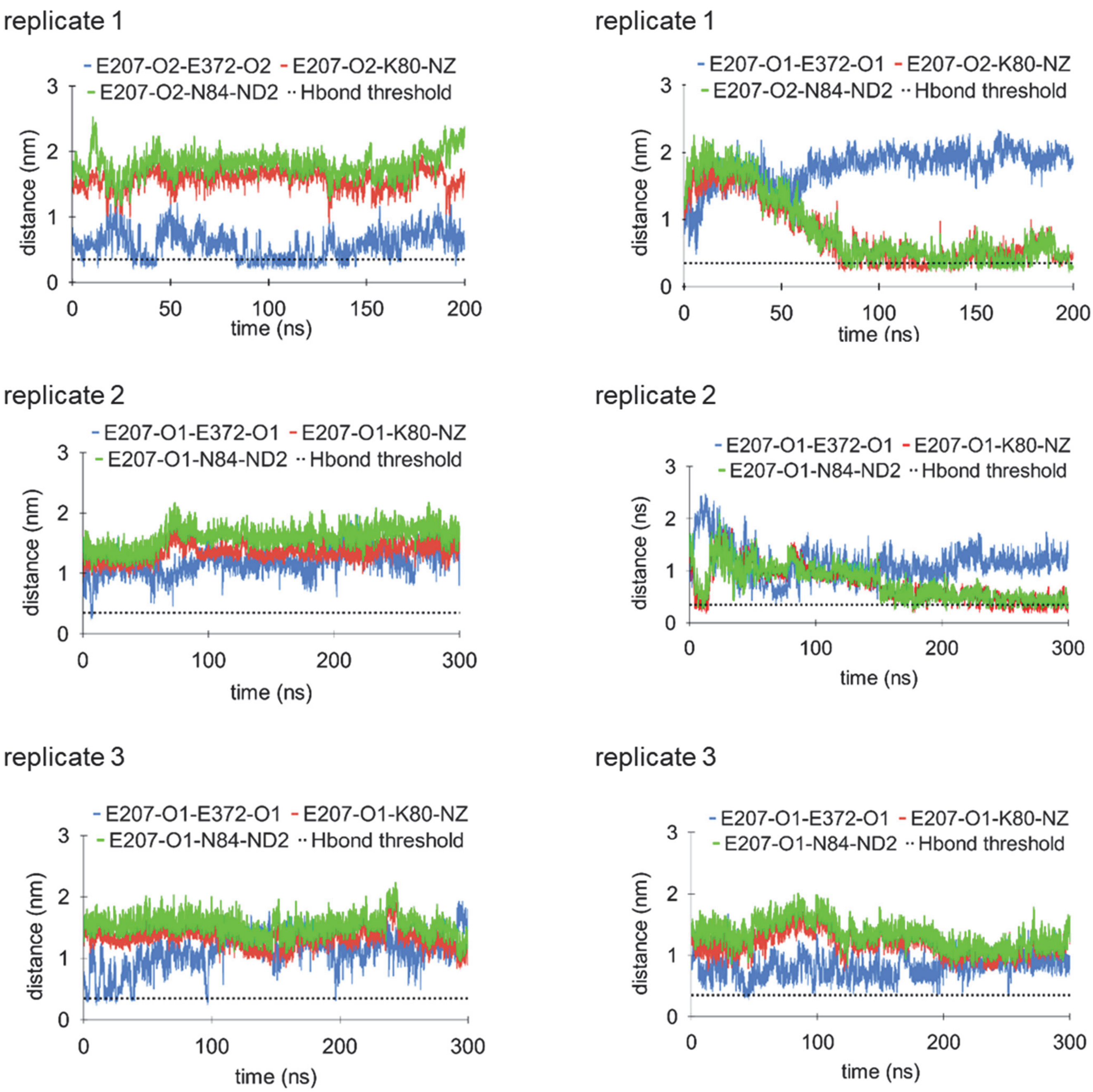

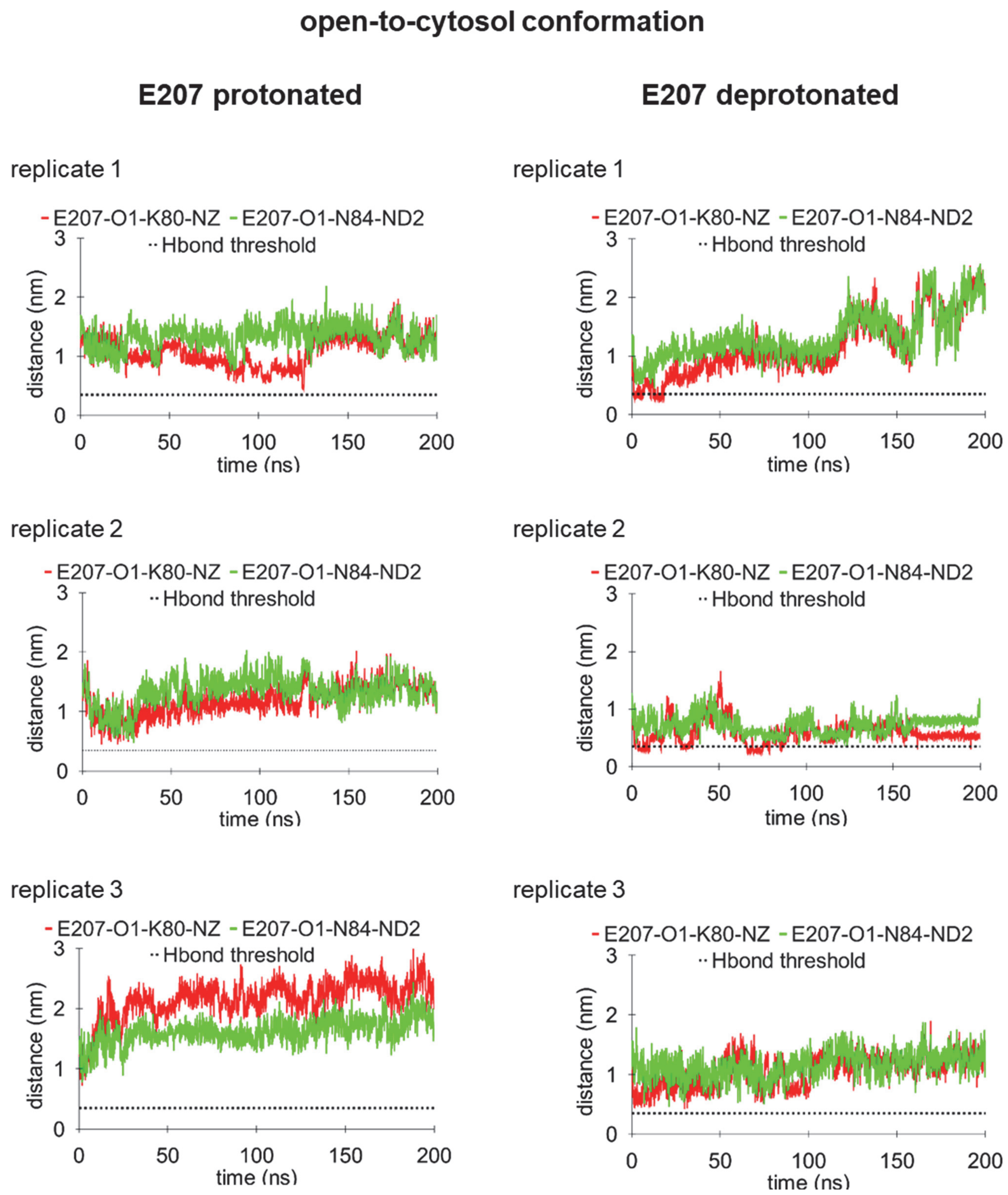

Supplementary Figure 14 I Distance between hydrogen bond donors and acceptors, E207 and E372/K80/N84, in MD simulations of the open-to-vacuole, occluded, and open-to-cytoplasm conformation of PfCRT^{Dd2}, with E207 on the protonated and deprotonated form. The results of three independent MD simulations per condition are shown. Black dotted lines indicate the threshold distance for the formation of a hydrogen bond of 3.5 Å.

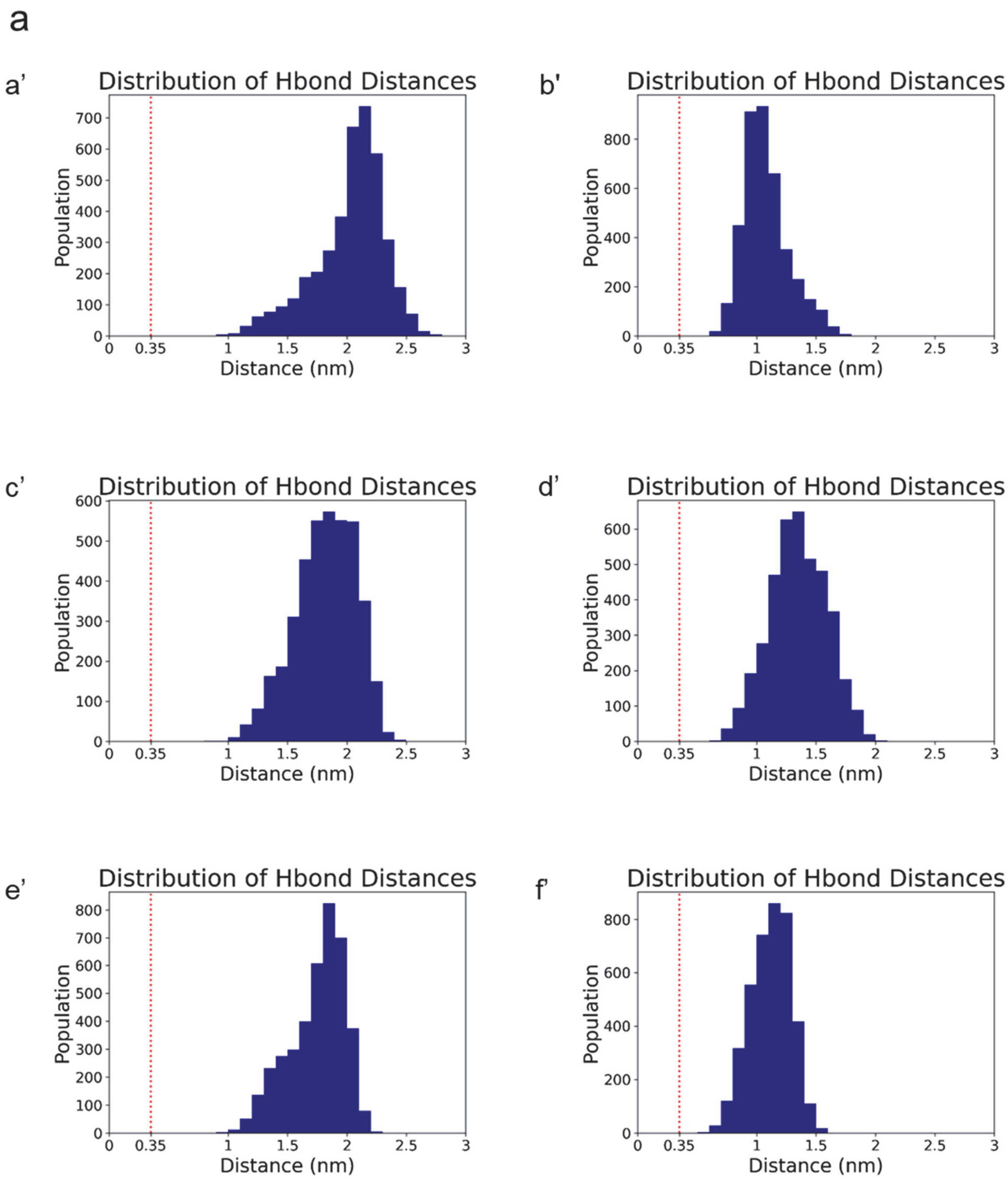

Supplementary Figure 15a I Distribution of distances between hydrogen bond donors and acceptors, E207 and K80/E372, in MD simulations of the open-to-vacuole conformation, E207 protonated. Red dotted lines indicate the threshold distance for the formation of a hydrogen bond. a', E207-E372 in replica 1. b', E207-K80 in replica 1.c', E207-E372 in replica 2.d', E207-K80 in replica 2. e', E207-E372 in replica 3. f', E207-K80 in replica 3.

b

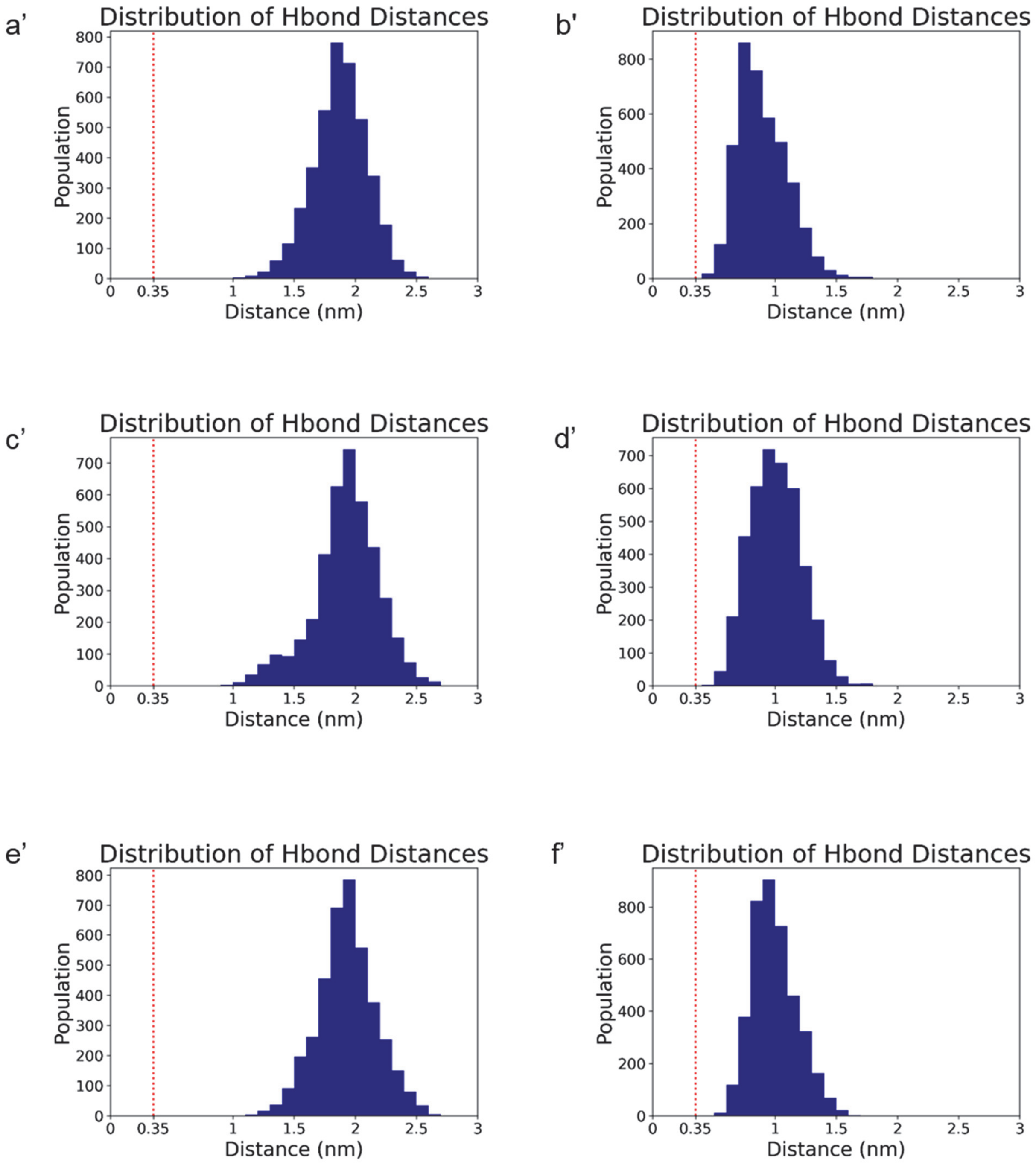

Supplementary Figure 15b I Distribution of distances between hydrogen bond donors and acceptors, E207 and K80/E372, in MD simulations of the open-to-vacuole conformation, E207 deprotonated. Red dotted lines indicate the threshold distance for the formation of a hydrogen bond. a’, E207-E372 in replica 1. b’, E207-K80 in replica 1.c’, E207-E372 in replica 2.d’, E207-K80 in replica 2. e’, E207-E372 in replica 3. f’, E207-K80 in replica

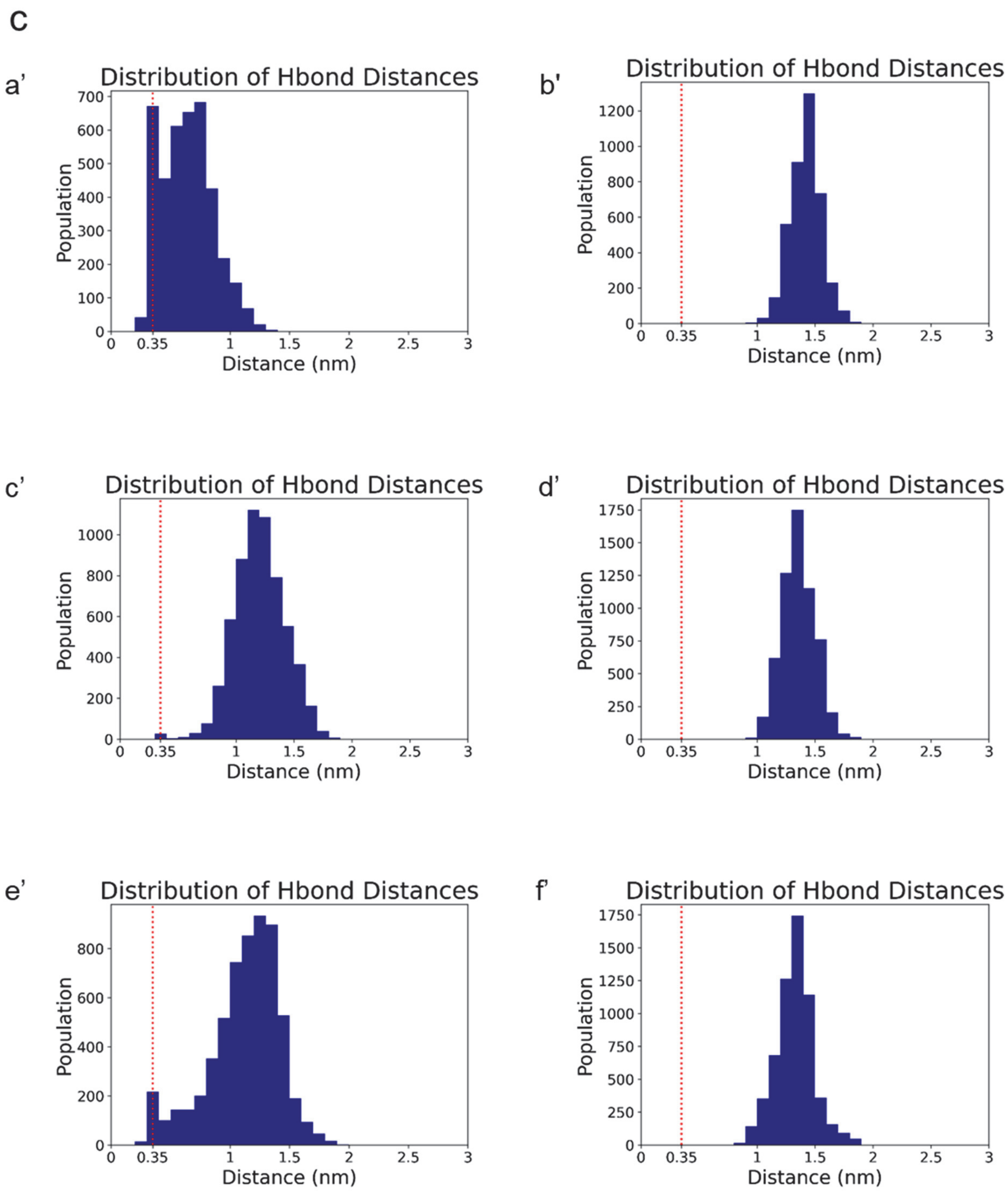

Supplementary Figure 15c I Distribution of distances between hydrogen bond donors and acceptors, E207 and K80/E372, in MD simulations of the occluded conformation, E207 protonated. Red dotted lines indicate the threshold distance for the formation of a hydrogen bond. a’, E207-E372 in replica 1. b’, E207-K80 in replica 1. c’, E207-E372 in replica 2. d’, E207-K80 in replica 2. e’, E207-E372 in replica 3. f’, E207-K80 in replica 3.

d

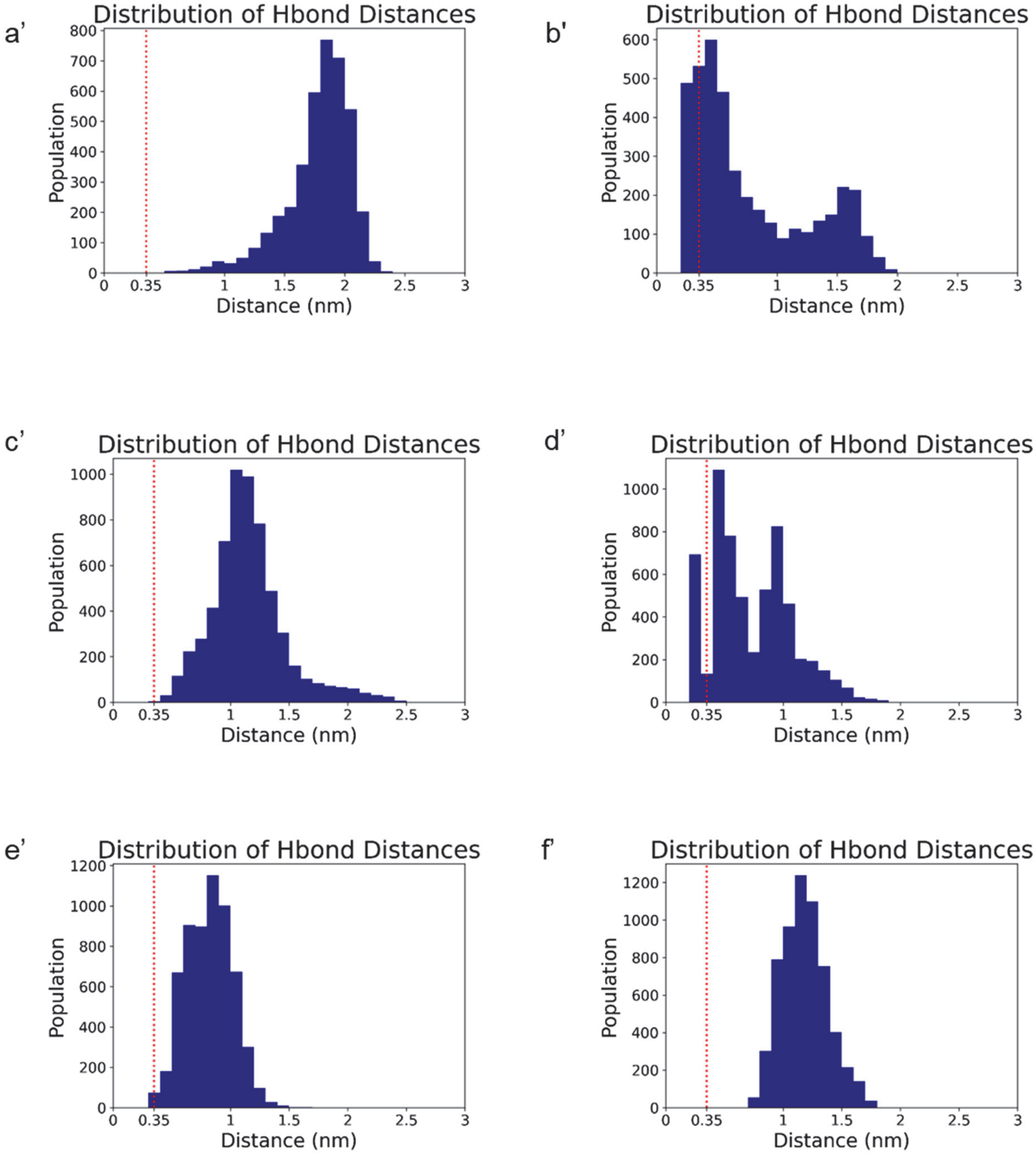

Supplementary Figure 15d I Distribution of distances between hydrogen bond donors and acceptors, E207 and K80/E372, in MD simulations of the occluded conformation, E207 deprotonated. Red dotted lines indicate the threshold distance for the formation of a hydrogen bond. a', E207-E372 in replica 1. b', E207-K80 in replica 1. c', E207-E372 in replica 2. d', E207-K80 in replica 2. e', E207-E372 in replica 3. f', E207-K80 in replica 3.

e

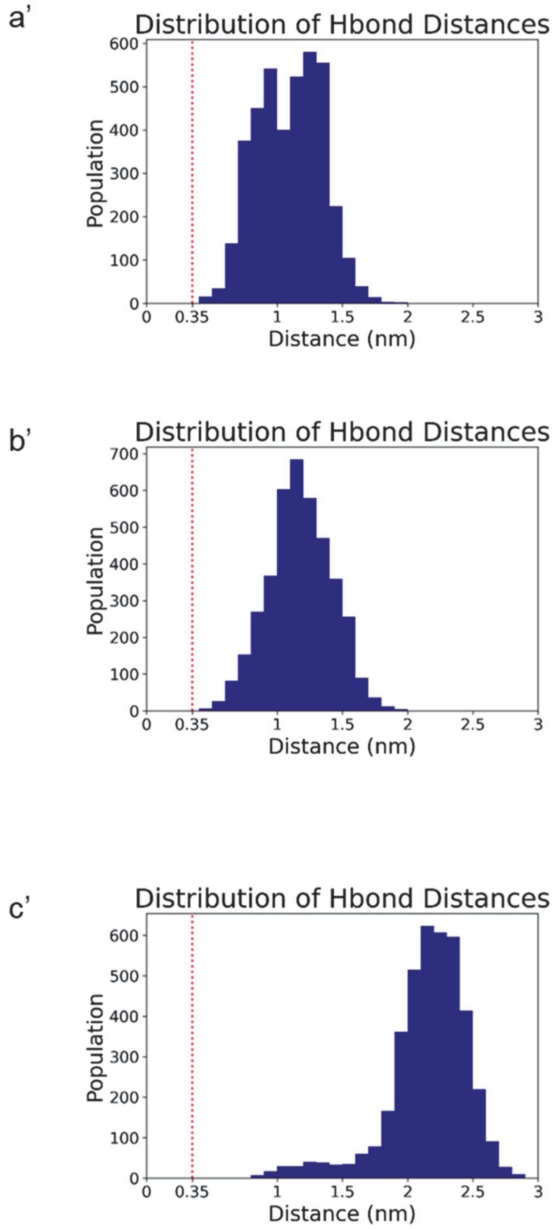

Supplementary Figure 15e I Distribution of distances between hydrogen bond donors and acceptors, E207 and K80, in MD simulations of the open-to-cytosol conformation, E207 protonated. Red dotted lines indicate the threshold distance for the formation of a hydrogen bond. a’, E207-K80 in replica 1. b’, E207-K80 in replica 2. c’, E207-K80 in replica 3.

f

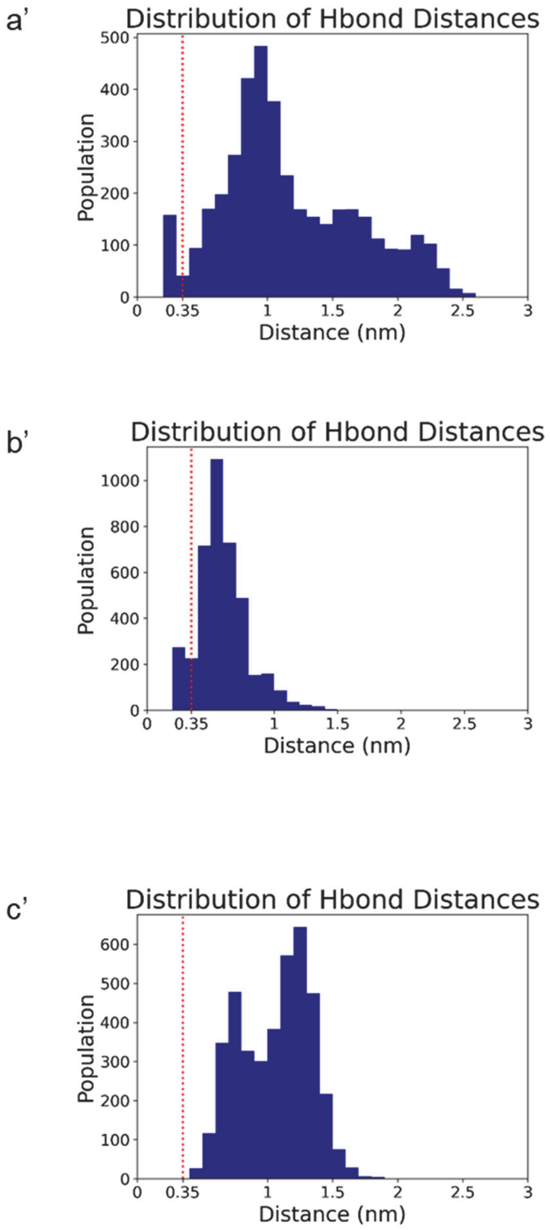

Supplementary Figure 15f I Distribution of distances between hydrogen bond donors and acceptors, E207 and K80, in MD simulations of the open-to-cytosol conformation, E207 deprotonated. Red dotted lines indicate the threshold distance for the formation of a hydrogen bond. a', E207-K80 in replica 1. b', E207-K80 in replica 2. c', E207-K80 in replica 3.

ATGAAGTTCG CCTCTAAGAA GAACAATCAA AAGAACTCCT CCAAGAATGC TGAAAGAGCT 60

AGAGCTGCTG ATAATGCTGC TCAAGAAGGT AACGGTTCTA GATTGGGTGG TGGTTCTTGT 120

TTGGGTAAAT GTGCTCATGC TGCTAAAGCT GCCTTCAAAG AAATCAAGGA CAACATCTTC 180

ATCTACATCT TGTCCATCAT CTACTTGTCC GTTTGCGTTA TTGAAACCAT CTTCGCCAAG 240

AGAACCTTGA ACAAGATTGG TAACTACTCT TTCGTTACCT CTGAAACCCA TAACTTCATC 300

TGCATGATCA TGTTCTTCAT CGTCTATTCC TTGTTCGGTA ACAAGAAGGG TAACTCCAAA 360

GAAAGACACA GATCCTTCAA CTTGCAATTC TTCGCCATTT CTATGTTGGA TGCCTGCTCT 420

GTTATTTTGG CTTTCATCGG TTTGACTAGA ACTACCGGTA ACATCCAATC TTTCGTCTTG 480

CAATTGTCCA TTCCAATCAA TATGTTCTTC TGCTTCTTGA TCTTGAGATA CAGATACCAC 540

TTGTACAATT ACTTGGGTGC CGTTATTATT GTCGTTACCA TTGCCTTGGT TGAAATGAAG 600

TTGTCCTTCG AAACCCAAGA AGAAAACTCC ATCATCTTCA ACTTGGTTTT GATCTCCTCA 660

TTGATCCCAG TTTGTTTCTC TAACATGACC AGAGAAATCG TTTTCAAGAA GTACAAGATC 720

GACATCTTGA GATTGAACGC TATGGTTTCC TTCTTCCAAT TATTCACCTC CTGCTTGATT 780

TTGCCAGTTT ACACCTTGCC ATTCTTGAAA GAATTGCACT TGCCATACAA CGAAATTTGG 840

ACCAACATCA AGAATGGTTT CGCTTGTTTG TTCTTGGGTA GAAACACCGT TGTTGAAAAC 900

TGTGGTTTGG GTATGGCTAA GTTGTGTGAT GATTGTGATG GTGCTTGGAA AACTTTCGCT 960

TTGTTCTCCT TCTTCTCCAT TTGCGATAAC TTGATCACCT CCTACATTAT CGATAAGTTC 1020

TCCACTATGA CCTACACTAT CGTATCTTGC ATTCAAGGTC CAGCTACTGC TATTGCTTAC 1080

TACTTCAAGT TCTTGGCTGG TGATGTTGTT ATTGAACCTA GATTATTGGA CTTCGTCACC 1140

TTGTTTGGTT ACTTGTTCGG TTCCATTATC TACAGAGTCG GTAACATCAT CTTGGAAAGA 1200

AAGAAGATGA GAAACGAAGA AAACGCTGAT TCTGCTGGTG CTTTGACTAA TGTTGATTCT 1260

GCTGCTACTC AACCTAGG

Supplementary Figure 16 I Codon-optimized and oocyte-adapted sequence of PfCRT^{Dd2}, according to Martin et al. (2009) [1].

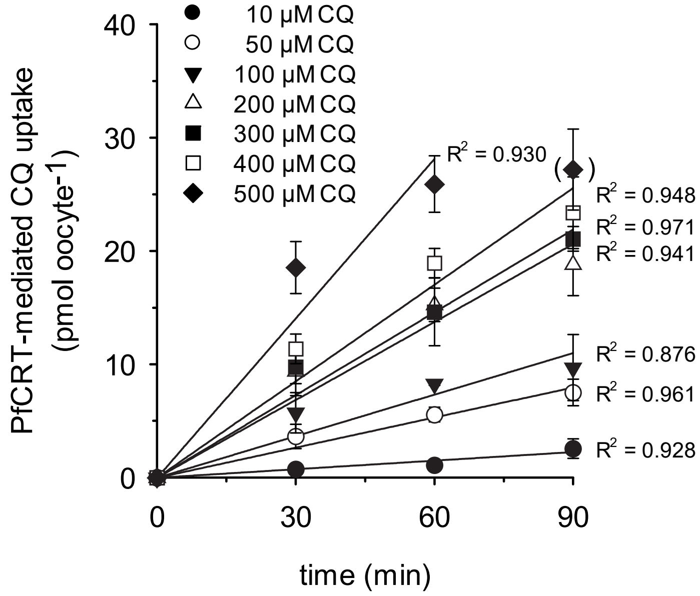

Supplementary Figure 17 I Time courses of PfCRT^{Dd2}-mediated CQ uptake at the CQ concentrations indicated. The mean ± SEM of 4 biologically independent samples are shown per data point. Data points were fit using a linear regression and the R^2 value was calculated for each condition.

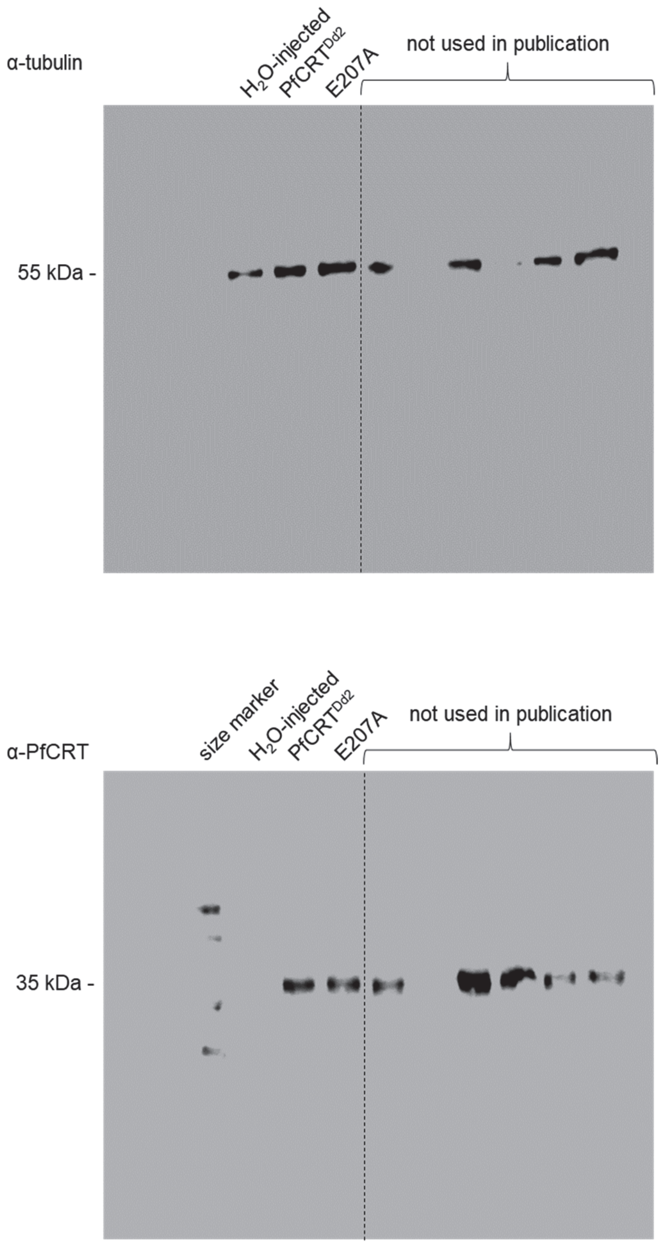

Supplementary Figure 18 I Uncropped Western blot images presented in Figure 2b.

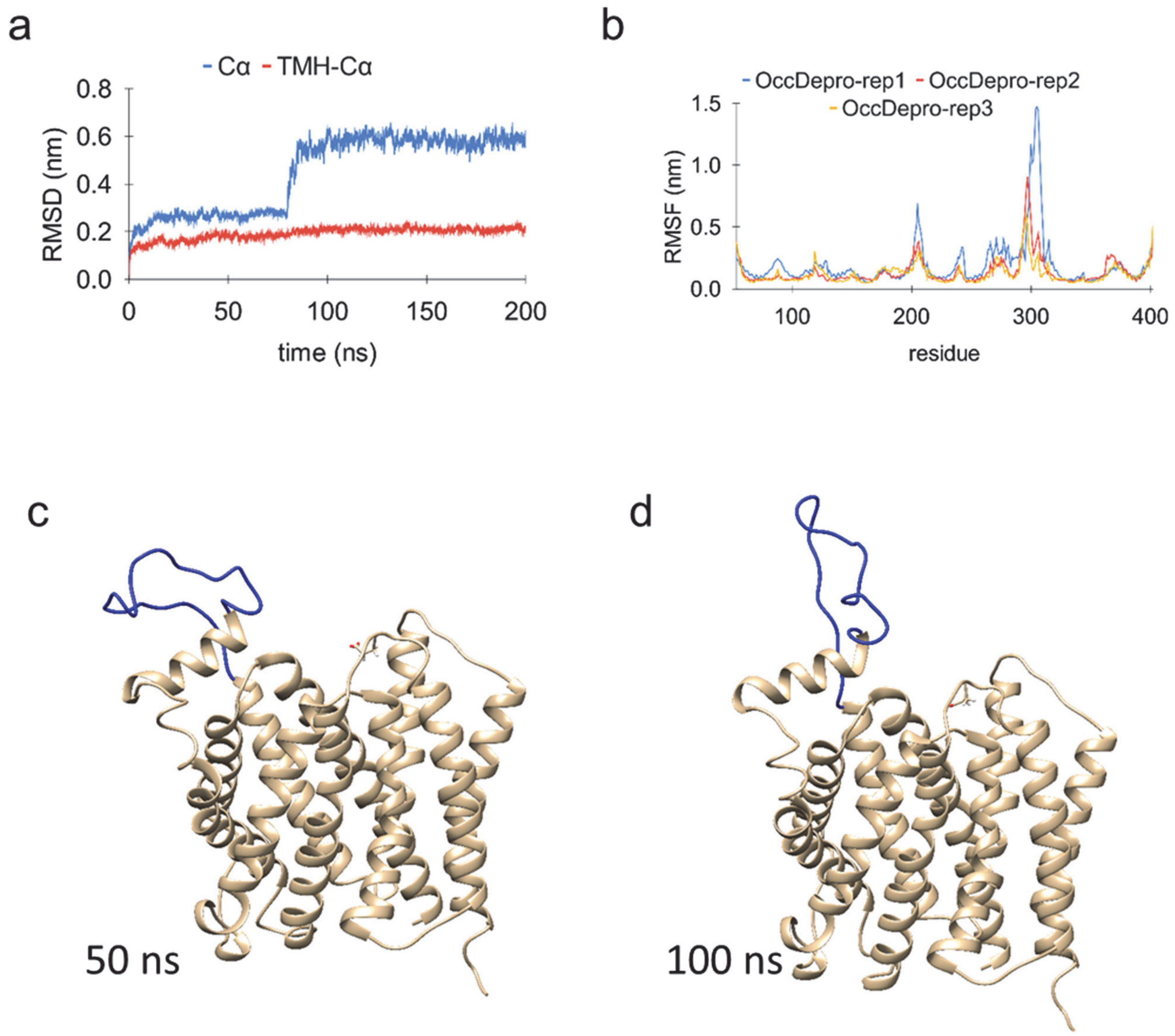

Supplementary Figure 19 I Structural changes of a disordered region in one MD simulation (replica 1) of the occluded conformation, with E207 deprotonated. a, RMSD values of replica 1, **b**, comparison of RMSF values of all 3 replicas. **c** and **d**, structural changes of the residues around residue N300. The sudden rise in the RMSD values is the result of the structural changes in this region. Figures c and d are snapshots from 50 and 100 ns of the MD simulation.

Supplementary Table 1. Mean PfCRT-mediated CQ transport activity ± SEM at pH 6.0 and pH 4.5 and corresponding R-value. The R-value was calculated as the mean value of all R-values obtained in paired experiments. CD2 harbors the following mutations: D24A, D57A, Y179A, F235A, D241A, E406A, and D419A. CD3 harbors the following mutations: F3A, E29A, F52A, Y238A, F340A, E399A and E407A.

	PfCRT variant	Activity at pH 6.0 pmol h^{-1} $oocyte^{-1}$	Activity at pH 4.5 pmol h^{-1} $oocyte^{-1}$	R-value
1	$PfCRT^{Dd2}$	5.05 ± 0.23	1.20 ± 0.10	0.24 ± 0.01
2	E18A	2.60 ± 0.53	1.51 ± 0.34	0.57 ± 0.06
3	H46A	5.06 ± 0.47	2.03 ± 0.27	0.40 ± 0.03
4	E54A	3.36 ± 0.45	1.38 ± 0.17	0.44 ± 0.07
5	E75A	1.73 ± 0.06	0.89 ± 0.11	0.51 ± 0.05
6	Y89A	1.77 ± 0.09	0.73 ± 0.05	0.43 ± 0.06
7	E95A	2.74 ± 0.74	1.21 ± 0.20	0.41 ± 0.11
8	H97A	6.57 ± 0.71	2.71 ± 0.36	0.40 ± 0.04
9	E121A	3.95 ± 0.82	1.35 ± 0.09	0.43 ± 0.06
10	H123A	4.85 ± 0.43	1.96 ± 0. 28	0.36 ± 0.09
11	Y177A	2.94 ± 0.26	1.25 ± 0.21	0.44 ± 0.11
12	H180A	3.12 ± 0.12	1.14 ± 0.27	0.36 ± 0.09
13	E198A	3.10 ± 0.44	1.23 ± 0.15	0.44 ± 0.11
14	F203A	2.55 ± 0.14	0.93 ± 0.19	0.36 ± 0.07
15	E204A	2.98 ± 0.34	1.43 ± 0.06	0.51 ± 0.09
16	E207A	2.24 ± 0.15	2.04 ± 0.18	0.89 ± 0.03
17	E208A	5.43 ± 0.38	2.65 ± 0.40	0.48 ± 0.05
18	E232A	1.67 ± 0.12	1.07 ± 0.12	0.65 ± 0.06
19	H273A	2.43 ± 0.40	0.08 ± 0.04	0.03 ± 0.02
20	F268A	2.93 ± 0.28	1.06 ± 0.19	0.40 ± 0.10
21	E271A	1.75 ± 0.14	1.01 ± 0.09	0.61 ± 0.09
22	Y276A	3.51 ± 0.35	1.50 ± 0.12	0.45 ± 0.09
23	E278A	2.21 ± 0.24	1.17 ± 0.07	0.56 ± 0.10
24	W280A	2.64 ± 0.28	0.92 ± 0.11	0.35 ± 0.03
25	F287A	4.71 ± 0.24	1.50 ± 0.05	0.32 ± 0.02
26	F291A	3.23 ± 0.24	1.61 ± 0.31	0.52 ± 0.11
27	E299A	3.32 ± 0.54	1.87 ± 0.19	0.60 ± 0.12
28	D310A	1.25 ± 0.18	0.96 ± 0.15	0.77 ± 0.07
29	D311A	3.22 ± 0.61	1.07 ± 0.25	0.33 ± 0.05
30	D313A	4.55 ± 0.45	1.49 ± 0.21	0.33 ± 0.04
31	F319A	3.64 ± 0.45	0.90 ± 0.25	0.26 ± 0.07
32	F322A	1.05 ± 0.12	0.72 ± 0.12	0.68 ± 0.04
33	D338A	5.20 ± 0.52	2.35 ± 0.19	0.47 ± 0.06
34	D368A	3.05 ± 0.29	1.36 ± 0.32	0.44 ± 0.08
35	E372A	4.71 ± 0.40	2.23 ± 0.25	0.49 ± 0.08
36	R374A	3.81 ± 0.61	0.90 ± 0.14	0.23 ± 0.03
37	D377A	1.44 ± 0.13	0.62 ± 0.01	0.44 ± 0.04
38	D410A	5.09 ± 0.24	2.38 ± 0.18	0.47 ± 0.06
39	CD2	2.31 ± 0.23	1.26 ± 0.05	0.56 ± 0.05
40	CD3	2.73 ± 0.57	1.78 ± 0.46	0.62 ± 0.06
41	D137N	0.95 ± 0.18	0.57 ± 0.14	0.59 ± 0.04
42	D329N	1.25 ± 0.32	0.39 ± 0.14	0.32 ± 0.07

Supplementary Table 2. Cluster sizes for protein configurations from MD simulations and docking scores obtained for docking CQ to the center of each cluster. Protein configurations were obtained from 3 independent 200-ns MD simulations for each conformation of PfCRT. MD simulations were performed for different conformations (Va: open-to-vacuole, Occ: occluded, Cy: open-to-cytoplasm) and different protonation states for E207 (Pro: protonated; Depro: deprotonated). Clustering was performed using Gromacs and the gromos method. Docking was performed using AutoDock Vina.

System	Cluster Number	Cluster Size	score (kcal/mol)
VaPro	1	13/45	-6.50
VaPro	2	13/45	-5.71
VaPro	3	10/45	-5.97
VaPro	4	4/45	-5.96
VaPro	5	2/45	-5.55
VaPro	6	2/45	-5.35
VaPro	7	1/45	-5.97
VaPro	8	1/45	-5.93
VaDepro	1	15/45	-5.47
VaDepro	2	13/45	-5.66
VaDepro	3	7/45	-5.14
VaDepro	4	4/45	-5.47
VaDepro	5	2/45	-5.91
VaDepro	6	2/45	-6.07
VaDepro	7	1/45	-5.63
VaDepro	8	1/45	-5.72
VaDepro	9	1/45	-5.78
OccPro	1	16/45	-6.49
OccPro	2	12/45	-5.91
OccPro	3	9/45	-6.50

OccPro	4	4/45	-5.96
OccPro	5	3/45	-5.58
OccPro	6	2/45	-5.57
OccDepro	1	14/45	-5.50
OccDepro	2	11/45	-4.80
OccDepro	3	11/45	-6.42
OccDepro	4	4/45	-6.68
OccDepro	5	3/45	-5.45
OccDepro	6	2/45	-5.06
OccDepro	7	1/45	-6.51
CyPro	1	15/45	-5.12
CyPro	2	14/45	-4.90
CyPro	3	8/45	-4.71
CyPro	4	8/45	-4.49
CyPro	5	1/45	-4.51
CyDepro	1	15/45	-5.32
CyDepro	2	15/45	-5.28
CyDepro	3	11/45	-5.80
CyDepro	4	3/45	-5.01
CyDepro	5	1/45	-6.45
CyDepro	6	1/45	-5.89

Supplementary Table 3. Length and quality metrics for the models of the three different conformations of PfCRT^{Dd2} (open-to-vacuole, occluded, open-to-cytoplasm). As a reference, the quality assessment of PDB 6UKJ and PDB 5Y79, which were used as templates to build the models, are also provided. The quality metrics were obtained using MolProbity, as implemented in the SWISS-MODEL web server [2,3].

model	residues	Ramachandran favored (%)	Ramachandran outliers (%)	Rotamer outliers (%)	MolProbity score[a]
PfCRT (open-to-vacuole)	47-405	95.31	0.31	3.86	2.48
PfCRT (occluded)	53-402	96.13	0.97	4.25	1.24
PfCRT (open-to- cytoplasm)	59-361	87.36	4.46	8.30	1.81
PDB 6UKJ	47-405	96.53	0	0.96	1.43
PDB 5Y79	100-404	99.34	0	1.39	1.43

[a]Quality score which represents the crystallographic resolution at which such a quality would be expected.

Supplementary Table 4a. Average, standard deviation (STD), minimum (min) and maximum (max) pKa values predicted for titratable residues of PfCRT^{Dd2} in the open-to-vacuole conformation. Protein configurations for calculations were obtained from three independent MD simulations, with E207 protonated or deprotonated. Predictions were done with Propka 3.5.

	open-to-vacuole, E207 protonated				open-to-vacuole, E207 deprotonated			
Residues	**Average**	**STD**	**min**	**max**	**Average**	**STD**	**min**	**max**
ASP 57	3.28	0.44	2.81	4.24	3.18	0.53	1.83	4.06
ASP 137	3.41	0.32	2.84	4.15	3.26	0.32	2.86	4.27
ASP 241	3.75	0.67	1.71	4.76	4.08	0.45	2.64	4.69
ASP 310	3.07	0.28	2.50	3.75	3.39	0.76	1.59	4.99
ASP 311	3.41	0.46	2.83	4.20	3.05	0.52	2.25	4.79
ASP 313	3.83	0.19	2.80	4.03	3.77	0.57	2.10	4.72
ASP 329	4.51	0.81	3.65	6.13	4.82	0.73	3.63	6.21
ASP 338	3.90	0.70	2.35	5.19	3.92	0.59	2.24	5.08
ASP 368	3.40	0.49	2.33	4.05	3.31	0.52	1.98	4.04
ASP 377	3.77	0.57	2.83	4.64	3.71	0.57	2.61	4.56
GLU 54	2.89	0.80	1.41	4.14	3.19	0.88	1.63	4.97
GLU 75	8.73	1.13	5.96	10.79	8.76	1.32	6.44	11.58
GLU 95	6.23	0.48	5.15	6.91	5.89	0.45	4.88	6.80
GLU 121	3.62	0.79	1.63	4.61	3.24	0.66	2.09	4.62
GLU 198	6.27	0.50	5.43	7.23	6.26	0.53	5.27	8.15
GLU 204	3.39	1.02	1.67	4.59	3.70	0.92	1.71	4.71
GLU 207	4.43	0.38	3.76	5.18	4.19	0.62	3.21	5.60
GLU 208	3.32	0.72	1.80	4.94	4.06	0.60	2.79	4.69
GLU 232	5.37	0.43	4.51	6.53	5.29	0.64	3.15	7.15
GLU 271	4.01	0.52	2.60	4.81	3.81	1.00	1.87	5.32
GLU 278	4.45	0.39	3.24	4.92	4.21	0.49	3.15	4.96

GLU 299	3.93	0.72	2.06	4.65	4.20	0.63	2.68	5.02
GLU 372	4.43	0.34	3.59	4.72	4.41	0.38	3.46	4.71
HIS 97	4.49	0.29	4.05	5.44	4.60	0.18	4.00	5.18
HIS 123	6.28	0.47	5.60	7.28	6.14	0.30	5.45	6.81
HIS 180	6.28	0.21	6.06	7.10	6.22	0.12	6.07	6.88
HIS 273	6.67	0.15	6.27	6.91	6.54	0.25	5.81	6.90
CYS 72	12.19	0.59	10.90	13.91	12.08	0.54	10.80	13.49
CYS 101	11.53	0.28	10.68	12.04	11.45	0.25	10.85	11.90
CYS 139	9.78	0.11	9.57	10.08	9.78	0.13	9.58	10.08
CYS 171	12.38	1.88	7.76	16.51	12.04	1.84	7.96	16.25
CYS 225	11.27	1.71	7.75	15.70	11.46	1.72	7.89	16.27
CYS 258	10.22	0.41	9.30	11.05	9.99	0.46	9.27	10.99
CYS 289	99.99	0.00	99.99	99.99	99.99	0.00	99.99	99.99
CYS 301	99.99	0.00	99.99	99.99	99.99	0.00	99.99	99.99
CYS 309	99.99	0.00	99.99	99.99	99.99	0.00	99.99	99.99
CYS 312	99.99	0.00	99.99	99.99	99.99	0.00	99.99	99.99
CYS 328	10.12	0.16	9.83	10.47	10.17	0.20	9.83	10.72
CYS 350	11.62	0.32	10.84	12.50	11.97	0.40	10.95	12.63
TYR 62	11.13	0.49	10.44	12.10	11.07	0.36	10.50	12.09
TYR 68	18.44	0.72	16.99	20.07	18.09	1.23	15.29	21.09
TYR 89	12.84	0.34	12.14	13.43	12.21	0.69	10.80	13.35
TYR 109	10.34	0.70	8.84	12.59	10.44	0.52	9.22	11.87
TYR 177	11.74	0.81	10.46	13.52	11.87	1.09	9.74	14.66
TYR 179	13.27	0.68	12.21	15.48	13.37	0.54	12.22	14.58
TYR 182	10.14	0.02	10.10	10.21	10.14	0.03	10.09	10.22
TYR 184	10.30	0.09	10.15	10.57	10.36	0.09	10.22	10.77
TYR 238	10.06	0.46	9.15	12.09	9.86	0.32	9.00	10.20

TYR 264	11.66	0.41	10.89	12.69	11.55	0.41	10.76	12.36
TYR 276	10.05	0.11	9.49	10.15	10.03	0.14	9.47	10.17
TYR 335	10.19	0.19	9.59	10.64	10.28	0.19	9.53	10.68
TYR 345	12.39	0.54	11.36	14.36	12.24	0.55	11.10	13.22
TYR 360	11.27	0.55	9.96	12.42	11.42	0.60	10.09	12.62
TYR 361	11.66	0.36	10.69	12.30	12.16	0.40	11.20	13.00
TYR 384	14.44	0.77	12.92	15.54	14.27	0.77	12.96	15.61
TYR 391	12.63	0.49	11.19	13.79	12.64	0.53	11.19	13.72
LYS 49	10.46	0.02	10.39	10.48	10.45	0.03	10.31	10.48
LYS 53	11.22	0.43	10.37	11.58	11.15	0.48	10.33	11.67
LYS 56	10.37	0.21	10.02	11.45	10.39	0.29	10.07	11.44
LYS 80	9.99	0.58	9.33	11.47	10.21	0.54	9.40	11.43
LYS 85	10.99	0.74	9.74	12.38	11.17	0.61	9.65	12.32
LYS 115	10.42	0.24	9.68	11.16	10.43	0.13	10.01	10.84
LYS 116	10.38	0.23	9.92	11.34	10.30	0.25	9.57	11.00
LYS 120	10.43	0.13	9.92	11.09	10.40	0.17	9.75	10.63
LYS 200	10.90	0.44	10.26	11.47	10.83	0.43	10.37	11.48
LYS 236	10.47	0.31	9.87	11.36	10.55	0.39	9.81	11.83
LYS 237	10.76	0.40	10.28	12.32	10.72	0.30	10.20	11.46
LYS 239	10.32	0.32	9.73	11.36	10.32	0.17	10.05	11.20
LYS 270	10.74	0.45	10.06	12.28	10.70	0.47	10.06	12.43
LYS 284	10.26	0.07	10.09	10.39	10.27	0.08	10.02	10.40
LYS 307	10.63	0.59	9.89	12.36	10.43	0.47	9.47	11.45
LYS 317	10.39	0.05	10.24	10.47	10.36	0.06	10.16	10.45
LYS 339	11.04	0.40	10.32	11.64	11.11	0.41	10.35	11.64
LYS 363	10.40	0.08	10.22	10.55	10.52	0.28	10.31	11.40
ARG 81	12.01	0.29	11.49	12.90	12.39	0.36	11.77	13.16

ARG 122	12.62	0.29	12.20	13.47	12.62	0.45	12.10	13.86
ARG 124	12.43	0.23	12.04	13.61	13.08	0.72	12.28	14.38
ARG 150	13.14	0.58	12.15	14.16	12.97	0.69	12.09	14.07
ARG 176	12.33	0.08	12.06	12.45	12.35	0.09	12.00	12.44
ARG 178	12.37	0.11	11.97	12.54	12.30	0.14	11.88	12.48
ARG 231	13.29	0.40	12.32	13.95	13.47	0.31	12.47	13.88
ARG 244	12.27	0.35	11.56	13.09	12.29	0.37	11.57	13.06
ARG 294	12.35	0.04	12.27	12.48	12.60	0.49	12.25	14.23
ARG 374	13.58	0.60	12.31	14.41	13.94	0.49	12.85	14.73
ARG 392	13.71	0.45	12.76	14.47	13.39	0.79	11.37	14.31

Supplementary Table 4b. Average, standard deviation (STD), minimum (min) and maximum (max) pKa values predicted for titratable residues of PfCRT^{Dd2} in the occluded conformation. Protein configurations for calculations were obtained from three independent MD simulations, with E207 protonated or deprotonated. Predictions were done with Propka 3.5.

	occluded, E207 protonated				occluded, E207 deprotonated			
Residue	**Average**	**STD**	**min**	**max**	**Average**	**STD**	**min**	**max**
ASP 57	3.43	0.42	2.26	4.12	3.36	0.41	2.62	4.03
ASP 137	3.40	0.34	2.84	4.37	3.60	0.61	2.74	5.05
ASP 241	2.57	0.70	1.21	4.61	2.57	0.70	1.43	3.70
ASP 310	3.42	0.52	2.35	4.13	3.81	0.36	2.79	4.21
ASP 311	2.87	0.49	1.99	3.76	3.04	0.51	1.79	4.04
ASP 313	3.81	0.51	2.38	4.35	3.54	0.45	2.72	4.12
ASP 329	5.98	0.38	5.14	6.89	6.06	0.84	5.14	11.10
ASP 338	3.42	0.65	2.12	4.89	4.03	0.60	2.59	5.49
ASP 368	3.77	0.45	2.04	4.07	3.92	0.02	3.86	3.99
ASP 377	3.61	0.70	2.13	4.85	3.22	0.67	2.11	4.49
GLU 54	4.45	0.27	3.85	4.83	4.30	0.37	3.27	4.86
GLU 75	8.86	0.70	7.75	10.38	9.44	1.42	6.09	13.64
GLU 95	6.96	0.41	6.18	8.15	6.42	0.60	5.07	7.36
GLU 121	3.43	0.75	1.97	4.60	4.17	0.52	2.92	4.71
GLU 198	4.99	0.49	3.83	5.97	4.55	0.46	3.64	5.72
GLU 204	3.36	0.66	1.76	4.68	4.17	0.64	2.82	5.38
GLU 207	5.30	0.52	4.19	6.78	3.79	0.82	2.34	5.27
GLU 208	4.82	0.61	3.33	5.69	4.24	0.49	3.12	4.88
GLU 232	5.96	0.88	4.04	8.17	7.42	0.42	6.46	8.41
GLU 271	4.21	0.51	3.05	4.88	4.18	0.47	2.96	4.72
GLU 278	4.25	0.59	2.79	5.26	4.55	0.25	4.01	4.89

GLU 299	4.70	0.50	3.51	5.74	3.86	0.63	2.53	4.60
GLU 372	4.32	0.48	2.98	4.76	4.12	0.60	2.23	5.05
HIS 97	3.48	0.36	2.87	4.33	3.92	0.49	2.95	4.92
HIS 123	5.83	0.43	4.87	6.64	5.55	0.43	4.75	6.64
HIS 180	6.25	0.23	5.89	6.79	6.22	0.44	5.66	7.13
HIS 273	6.31	0.07	6.09	6.39	6.30	0.32	5.56	7.31
CYS 72	14.25	0.87	12.51	15.75	14.64	1.00	11.02	15.92
CYS 101	12.48	0.79	10.86	14.10	12.59	0.61	11.47	14.03
CYS 139	9.69	0.40	8.96	10.52	9.95	0.25	9.17	10.35
CYS 171	10.83	0.44	9.75	11.70	11.12	0.31	10.25	11.61
CYS 225	11.85	0.38	10.51	12.42	12.18	0.22	11.70	12.68
CYS 258	12.40	0.48	10.90	13.16	12.10	0.51	10.70	13.22
CYS 289	99.99	0.00	99.99	99.99	99.99	0.00	99.99	99.99
CYS 301	99.99	0.00	99.99	99.99	99.99	0.00	99.99	99.99
CYS 309	99.99	0.00	99.99	99.99	99.99	0.00	99.99	99.99
CYS 312	99.99	0.00	99.99	99.99	99.99	0.00	99.99	99.99
CYS 328	10.32	0.30	9.77	10.91	10.50	0.25	10.14	11.12
CYS 350	10.68	0.53	9.78	11.59	11.03	0.30	10.30	11.94
TYR 62	10.15	0.11	9.76	10.42	10.17	0.13	9.43	10.48
TYR 68	15.79	1.10	13.96	19.61	15.02	0.80	12.77	16.70
TYR 89	11.27	0.43	10.35	12.16	10.92	0.43	10.22	12.17
TYR 109	10.46	0.39	9.78	11.20	10.11	0.30	9.15	10.91
TYR 177	10.83	0.57	10.21	12.12	10.72	0.45	10.12	12.48
TYR 179	10.54	0.46	10.03	11.59	10.61	0.44	10.02	11.47
TYR 182	10.59	0.42	10.10	11.43	10.49	0.35	10.13	11.32
TYR 184	10.23	0.12	10.08	10.52	10.29	0.15	10.07	10.68
TYR 238	10.27	1.04	8.50	12.98	11.88	1.72	8.41	14.45

TYR 264	11.45	0.69	10.19	12.65	11.40	0.61	10.30	12.79
TYR 276	10.21	0.18	9.52	10.56	10.63	0.85	10.04	12.71
TYR 335	10.27	0.42	9.50	11.33	10.68	0.28	10.09	11.29
TYR 345	14.26	1.01	12.39	15.91	14.84	0.41	13.95	15.76
TYR 360	12.63	0.44	11.70	13.52	12.69	0.51	11.67	13.56
TYR 361	10.91	0.44	10.26	11.76	11.06	0.83	10.11	13.73
TYR 384	12.94	0.64	11.41	14.96	12.72	0.74	11.31	13.91
TYR 391	11.78	0.96	10.66	13.73	12.53	0.99	10.83	14.28
LYS 56	10.45	0.17	10.32	11.12	10.45	0.10	10.35	11.13
LYS 80	8.50	0.29	7.90	9.14	8.54	0.49	7.47	10.03
LYS 85	9.87	0.42	8.70	10.41	9.62	0.37	8.69	10.30
LYS 115	10.33	0.06	10.19	10.43	10.28	0.11	9.96	10.41
LYS 116	10.40	0.14	10.06	10.66	10.56	0.22	10.06	11.34
LYS 120	10.34	0.12	10.02	10.63	10.38	0.11	10.04	10.51
LYS 200	11.10	0.46	10.26	11.76	10.97	0.52	10.32	12.29
LYS 236	10.60	0.23	9.86	11.26	10.48	0.26	9.81	11.28
LYS 237	10.51	0.37	9.95	11.41	10.12	0.23	9.34	10.40
LYS 239	10.60	0.40	10.05	11.34	10.46	0.24	10.08	11.23
LYS 270	10.62	0.32	10.36	11.55	10.80	0.42	10.36	11.55
LYS 284	10.83	0.77	9.78	12.40	10.87	0.48	9.79	11.53
LYS 307	10.93	0.40	10.12	11.58	10.76	0.49	10.22	11.64
LYS 317	10.41	0.03	10.25	10.45	10.51	0.25	10.29	11.47
LYS 339	10.96	0.47	9.83	12.05	10.63	0.37	10.10	11.50
LYS 363	10.15	0.26	9.72	11.20	10.10	0.20	9.55	10.31
ARG 81	12.13	0.52	11.41	14.11	12.06	0.14	11.74	12.29
ARG 122	12.58	0.40	11.98	13.88	12.34	0.08	12.16	12.47
ARG 124	12.39	0.41	11.68	14.17	12.39	0.60	11.51	13.47

ARG 150	12.27	0.26	12.03	13.93	12.45	0.51	11.79	13.93
ARG 176	12.43	0.03	12.34	12.48	12.44	0.03	12.34	12.52
ARG 178	12.45	0.25	12.32	14.13	12.46	0.20	12.25	13.25
ARG 231	13.27	0.31	12.23	13.82	12.70	0.89	10.67	13.78
ARG 244	12.88	0.67	11.44	14.17	12.62	0.33	11.92	13.43
ARG 294	12.47	0.29	12.25	13.64	12.25	0.46	11.41	13.88
ARG 374	13.65	1.00	12.46	15.61	14.20	1.20	12.44	16.85
ARG 392	11.76	0.21	11.42	12.25	11.72	0.19	11.28	12.16

Supplementary Table 4c. Average, standard deviation (STD), minimum (min) and maximum (max) pKa values predicted for titratable residues of PfCRT^{Dd2} in the open-to-cytoplasm conformation. Protein configurations for calculations were obtained from three independent MD simulations, with E207 protonated or deprotonated. Predictions were done with Propka 3.5.

	**open-to-cytoplasm E207 protonated**				**open-to-cytoplasm, E207 deprotonated**			
Residue	**Average**	**STD**	**min**	**max**	**Average**	**STD**	**min**	**max**
ASP 137	6.02	0.35	5.33	6.69	5.80	0.46	4.87	6.77
ASP 241	3.22	0.81	1.77	4.81	2.78	0.60	1.80	4.01
ASP 310	3.41	0.60	1.76	4.04	3.64	0.57	1.86	4.33
ASP 311	3.53	0.76	1.97	5.01	3.33	0.42	2.57	4.46
ASP 313	3.45	0.46	2.76	4.13	3.59	0.73	2.08	4.64
ASP 329	4.99	0.80	3.09	6.81	5.50	0.97	3.82	8.17
ASP 338	7.25	1.03	5.26	8.55	5.38	0.54	4.13	6.39
GLU 75	5.73	0.87	3.86	7.02	5.78	1.03	3.85	7.58
GLU 95	6.39	0.46	5.55	7.30	6.71	0.48	5.63	7.99
GLU 121	3.37	0.64	1.78	4.62	3.97	0.53	2.81	4.80
GLU 198	4.56	0.49	3.54	6.11	4.68	0.61	3.66	5.90
GLU 204	3.62	0.66	2.11	4.82	3.88	0.45	2.58	4.69
GLU 207	4.70	0.37	3.52	5.33	4.30	0.60	2.81	5.53
GLU 208	4.31	0.48	2.91	4.93	4.18	0.66	2.58	4.81
GLU 232	3.73	0.53	2.57	4.76	3.45	0.76	1.83	4.45
GLU 271	3.90	0.72	2.08	4.98	4.14	0.52	2.97	4.74
GLU 278	4.44	0.39	3.09	5.17	4.29	0.63	2.50	5.19
GLU 299	4.35	0.49	2.77	4.82	4.08	0.52	2.45	4.73
HIS 97	5.23	0.71	4.23	7.23	5.33	0.88	3.89	7.01
HIS 123	6.27	0.48	5.61	7.48	6.04	0.39	5.26	7.00

HIS 180	6.00	0.53	5.19	6.92	6.48	0.43	5.88	7.14
HIS 273	6.24	0.28	5.83	6.84	6.10	0.14	5.69	6.52
CYS 72	10.02	0.50	9.39	11.32	10.97	0.51	9.78	12.07
CYS 101	11.98	0.31	11.20	12.59	11.76	0.46	10.83	12.70
CYS 139	9.70	0.28	9.23	10.25	9.93	0.46	9.27	10.85
CYS 171	9.38	0.55	8.41	10.71	9.51	0.10	9.29	9.72
CYS 225	10.19	0.41	9.37	10.94	9.48	0.43	8.46	10.28
CYS 258	9.08	0.48	8.37	9.86	8.94	0.36	8.38	9.50
CYS 289	99.99	0.00	99.99	99.99	99.99	0.00	99.99	99.99
CYS 301	99.99	0.00	99.99	99.99	99.99	0.00	99.99	99.99
CYS 309	99.99	0.00	99.99	99.99	99.99	0.00	99.99	99.99
CYS 312	99.99	0.00	99.99	99.99	99.99	0.00	99.99	99.99
CYS 328	9.61	0.19	9.36	10.07	9.89	0.51	8.98	10.73
CYS 350	10.12	1.02	8.23	11.70	9.79	0.41	9.15	10.57
TYR 62	10.17	0.19	9.60	10.62	10.21	0.10	10.06	10.50
TYR 68	11.28	0.59	9.83	12.68	12.05	1.63	9.33	15.55
TYR 89	12.39	0.52	11.35	13.72	12.41	0.51	10.83	13.34
TYR 109	9.99	0.25	9.27	10.39	10.23	0.38	9.02	11.08
TYR 177	10.05	0.26	9.41	10.50	10.12	0.16	9.53	10.33
TYR 179	10.52	0.29	9.79	11.23	10.25	0.23	9.53	10.68
TYR 182	10.52	0.61	9.66	11.94	10.29	0.31	9.20	10.91
TYR 184	12.11	0.50	11.11	13.52	10.65	0.38	9.75	11.29
TYR 238	10.93	0.45	10.10	11.96	10.52	0.75	9.19	11.99
TYR 264	12.41	0.93	10.22	13.92	11.21	0.52	10.45	12.51
TYR 276	10.25	0.39	9.47	11.07	10.20	0.18	9.85	10.70
TYR 335	10.15	0.36	9.20	10.86	10.19	0.33	9.49	11.48
TYR 345	10.42	0.34	10.07	11.50	10.40	0.38	9.54	11.32

LYS 80	9.92	0.50	8.73	11.28	10.11	0.33	8.54	10.58
LYS 85	10.20	0.12	9.89	10.37	10.23	0.14	9.80	10.44
LYS 115	10.34	0.31	9.45	11.32	10.50	0.31	9.87	11.52
LYS 116	10.48	0.14	10.20	11.25	10.52	0.22	10.18	11.15
LYS 120	10.46	0.27	10.02	11.32	10.41	0.15	10.02	11.09
LYS 200	10.88	0.43	10.35	11.60	10.94	0.36	10.37	11.48
LYS 236	10.38	0.07	10.20	10.57	10.31	0.40	9.69	11.45
LYS 237	10.67	0.34	10.25	11.28	10.66	0.42	9.83	11.48
LYS 239	10.49	0.57	9.84	11.33	10.42	0.28	9.96	11.42
LYS 270	10.52	0.25	10.27	11.51	10.61	0.36	10.25	11.60
LYS 284	11.24	0.61	9.80	12.33	10.74	0.49	9.70	12.31
LYS 307	10.63	0.41	9.92	11.45	10.61	0.40	10.16	11.68
LYS 317	10.42	0.03	10.32	10.49	10.39	0.08	10.15	10.48
LYS 339	9.70	0.40	8.71	10.67	10.06	0.53	9.13	11.38
ARG 81	12.30	0.06	12.15	12.42	12.32	0.05	12.22	12.41
ARG 122	12.65	0.55	12.02	13.96	12.44	0.50	11.31	13.61
ARG 124	13.03	0.73	11.87	14.27	12.11	0.49	11.19	13.44
ARG 150	12.67	0.71	11.33	14.21	12.75	0.72	11.34	14.27
ARG 176	12.48	0.16	12.33	13.24	12.45	0.15	12.22	12.96
ARG 178	12.48	0.13	12.34	12.97	12.49	0.25	12.27	13.52
ARG 231	12.06	0.54	11.02	13.07	11.98	0.67	10.63	13.42
ARG 244	12.84	0.47	12.09	13.96	13.12	0.68	12.24	14.23
ARG 294	12.42	0.45	11.82	14.26	12.93	0.60	12.32	14.29

Supplementary Table 5. Mean PfCRT-mediated CQ transport activity ± SEM at pH 6.0 and pH 4.5 and corresponding R-value of E207 substitution mutants and residues interacting with E207. The R-value was calculated as the mean value of all R-values obtained in paired experiments.

	PfCRT variant	Activity at pH 6.0 pmol h^{-1} $oocyte^{-1}$	Activity at pH 4.5 pmol h^{-1} $oocyte^{-1}$	R-value
1	$PfCRT^{Dd2}$	5.05 ± 0.23	1.20 ± 0.10	0.24 ± 0.01
2	E207D	5.13 ± 0.37	1.82 ± 0.06	0.36 ± 0.05
3	E207K	2.34 ± 0.24	1.91 ± 0.10	0.83 ± 0.04
4	E207L	2.25 ± 0.22	2.10 ± 0.17	0.95 ± 0.04
5	E207M	2.24 ± 0.41	1.85 ± 0.27	0.85 ± 0.02
6	E207Q	2.41 ± 0.53	2.15 ± 0.52	0.88 ± 0.01
7	E207Y	1.84 ± 0.21	1.70 ± 0.21	0.92 ± 0.06
8	E207C	1.53 ± 0.16	1.30 ± 0.11	0.86 ± 0.09
9	E207F	3.27 ± 0.42	2.67 ± 0.27	0.84 ± 0.09
10	E207H	4.64 ± 0.21	1.99 ± 0.16	0.43 ± 0.03
11	E207N	2.81 ± 0.43	2.54 ± 0.34	0.93 ± 0.07
12	E207R	2.69 ± 0.23	2.23 ± 0.16	0.84 ± 0.04
13	E207W	3.18 ± 0.35	2.40 ± 0.17	0.78 ± 0.06
14	E207S	3.07 ± 0.23	2.94 ± 0.21	0.96 ± 0.01
15	E207T	2.62 ± 0.31	2.41 ± 0.40	0.91 ± 0.05
16	E207G	2.55 ± 0.15	2.26 ± 0.46	0.88 ± 0.06
17	E207I	2.15 ± 0.11	2.05 ± 0.22	0.95 ± 0.01
18	E207P	2.45 ± 0.21	2.31 ± 0.3	0.93 ± 0.05
19	E207V	2.09 ± 0.15	2.0 ± 0.13	0.96 ± 0.02
20	E207A	2.24 ± 0.15	1.98 ± 0.18	0.89 ± 0.03
21	E207K/K80E	4.75 ± 0.91	1.85 ± 0.84	0.35± 0.10
22	K80A	2.26 ± 0.12	1.71 ± 0.08	0.76 ± 0.04
23	N84A	3.24 ± 0.32	1.41 ± 0.23	0.42 ± 0.04

Supplementary Table 6. Primers used in this study. Please note that a codon-optimized and oocyte-adapted version of PfCRT^{Dd2} was used, according to Martin et al. (2009) [1] (Supplementary Figure 16).

N°	Name	Sequence
1	5´globin for	GCAGAAGCTCAGAATAAACG
2	3´globin rev	GTAGCTTAGAGACTCCATTCG
3	PfCRTga H46A for	GTAAATGTGCTGCTGCTGCTAAAG
4	PfCRTga H46A rev	CTTTAGCAGCAGCAGCACATTTAC
5	PfCRTga H97A for	CTGAAACCGCTAACTTCATCTGC
6	PfCRTga H97A rev	GCAGATGAAGTTAGCGGTTTCAG
7	PfCRTga H273A for	GAAAGAATTGGCCTTGCCATAC
8	PfCRTga H273A rev	GTATGGCAAGGCCAATTCTTTC
9	PfCRTga H123A for	CCAAAGAAAGAGCCAGATCCTTC
10	PfCRTga H123A rev	GAAGGATCTGGCTCTTTCTTTGG
11	PfCRTga H180A for	GATACAGATACGCTTTGTACAATTAC
12	PfCRTga H180A rev	GTAATTGTACAAAGCGTATCTGTATC
13	PfCRTga D137N for	ATTTCTATGTTGAATGCCTGCTCTG
14	PfCRTga D137N rev	AGAGCAGGCATTCAACATAGAAATG
15	PfCRTga D329N for	TTCTCCATTTGCAATAACTTGATCACCTC
16	PfCRTga D329N rev	GAGGTGATCAAGTTATTGCAAATGGAGAAG
17	PfCRTga Y89A for	GATTGGTAACGCCTCTTTCGTTAC
18	PfCRTga Y89A rev	GTAACGAAAGAGGCGTTACCAATC
19	PfCRTga F203A for	GAAGTTGTCCGCCGAAACCCAAG
20	PfCRTga F203A rev	CTTGGGTTTCGGCGGACAACTTC
21	PfCRTga E204A for	GTTGTCCTTCGCTACCCAAGAAG
22	PfCRTga E204A rev	CTTCTTGGGTAGCGAAGGACAAC
23	PfCRTga E207A for	CGAAACCCAAGCTGAAAACTCC
24	PfCRTga E207A rev	GGAGTTTTCAGCTTGGGTTTCG
25	PfCRTga E208A for	CCCAAGAAGCTAACTCCATCATC
26	PfCRTga E208A rev	GATGATGGAGTTAGCTTCTTGGG
27	PfCRTga F268A for	CCTTGCCAGCCTTGAAAGAATTGC
28	PfCRTga F268A rev	GCAATTCTTTCAAGGCTGGCAAGG
29	PfCRTga E271A for	CATTCTTGAAAGCCTTGCACTTG
30	PfCRTga E271A rev	CAAGTGCAAGGCTTTCAAGAATG
31	PfCRTga Y276A for	CACTTGCCAGCCAACGAAATTTG
32	PfCRTga Y276A rev	CAAATTTCGTTGGCTGGCAAGTG
33	PfCRTga E278A for	GCCATACAACGCCATTTGGACC
34	PfCRTga E278A rev	GGTCCAAATGGCGTTGTATGGC
35	PfCRTga W280A for	CAACGAAATTGCTACCAACATC
36	PfCRTga W280A rev	GATGTTGGTAGCAATTTCGTTG
37	PfCRTga F287A for	CAAGAATGGTGCCGCTTGTTTG
38	PfCRTga F287A rev	CAAACAAGCGGCACCATTCTTG
39	PfCRTga F291A for	CGCTTGTTTGGCCTTGGGTAG
40	PfCRTga F291A rev	CTACCCAAGGCCAAACAAGCG
41	PfCRTga E299A for	CACCGTTGTTGCTAACTGTGG
42	PfCRTga E299A rev	CCACAGTTAGCAACAACGGTG

43	PfCRTga D310A for	GCTAAGTTGTGTGCTGATTGTG
44	PfCRTga D310A rev	CACAATCAGCACACAACTTAGC
45	PfCRTga D311A for	GTTGTGTGATGCTTGTGATGG
46	PfCRTga D311A rev	CCATCACAAGCATCACACAAC
47	PfCRTga D313A for	GTGATGATTGTGCTGGTGCTTG
48	PfCRTga D313A rev	CAAGCACCAGCACAATCATCAC
49	PfCRTga D368A for	GGCTGGTGCTGTTGTTATTG
50	PfCRTga D368A rev	CAATAACAACAGCACCAGCC
51	PfCRTga E372A for	GGTGATGTTGTTATTGCCCCTAG
52	PfCRTga E372A rev	CTAGGGGCAATAACAACATCACC
53	PfCRTga F319A for	CTTGGAAAACTGCCGCTTTGTTCTC
54	PfCRTga F319A rev	GAGAACAAAGCGGCAGTTTTCCAAG
55	PfCRTga F322A for	CTTTCGCTTTGGCCTCCTTCTTCTC
56	PfCRTga F322A rev	GAGAAGAAGGAGGCCAAAGCGAAAG
57	PfCRTga E75A for	GTTTGCGTTATTGCAACCATCTTC
58	PfCRTga E75A rev	GAAGATGGTTGCAATAACGCAAAC
59	PfCRTga E95A for	GTTACCTCTGCAACCCATAACTTC
60	PfCRTga E95A rev	GAAGTTATGGGTTGCAGAGGTAAC
61	PfCRTga E198A for	GCCTTGGTTGCAATGAAGTTG
62	PfCRTga E198A rev	CAACTTCATTGCAACCAAGGC
63	PfCRTga D377A for	CCTAGATTATTGGCCTTCGTCACC
64	PfCRTga D377A rev	GGTGACGAAGGCCAATAATCTAGG
65	PfCRTga E18A for	CCAAGAATGCTGCAAGAGCTAGAGC
66	PfCRTga E18A rev	GCTCTAGCTCTTGCAGCATTCTTGG
67	PfCRTga E54A for	CTGCCTTCAAAGCAATCAAGGACAAC
68	PfCRTga E54A rev	GTTGTCCTTGATTGCTTTGAAGGCAG
69	PfCRTga E121A for	GGTAACTCCAAAGCAAGACACAGATCC
70	PfCRTga E121A rev	GGATCTGTGTCTTGCTTTGGAGTTACC
71	PfCRTga Y177A for	CTTGATCTTGAGAGCCAGATACCACTTG
72	PfCRTga Y177A rev	CAAGTGGTATCTGGCTCTCAAGATCAAG
73	PfCRTga E232A for	CTAACATGACCAGAGCAATCGTTTTCAAG
74	PfCRTga E232A rev	CTTGAAAACGATTGCTCTGGTCATGTTAG
75	PfCRTga D338A for	CCTACATTATCGCTAAGTTCTCCAC
76	PfCRTga D338A rev	GTGGAGAACTTAGCGATAATGTAGG
77	PfCRTga D410A for	GAAAACGCTGCTTCTGCTGGTG
78	PfCRTga D410A rev	CACCAGCAGAAGCAGCGTTTTC
79	PfCRTga E207D for	CGAAACCCAAGACGAAAACTCC
80	PfCRTga E207D rev	GGAGTTTTCGTCTTGGGTTTCG
81	PfCRTga E121A for	GGTAACTCCAAAGCAAGACACAGATCT
82	PfCRTga E121A rev	AGATCTGTGTCTTGCTTTGGAGTTACC
83	PfCRTga E207M for	CGAGACCCAAATGGAAAACTCC
84	PfCRTga E207M rev	GGAGTTTTCCATTTGGGTCTCG
85	PfCRTga E207Y for	CGAGACCCAATACGAAAACTCC
86	PfCRTga E207Y rev	GGAGTTTTCGTATTGGGTCTCG
87	PfCRTga E207L for	CGAGACCCAACTAGAAAACTCC
88	PfCRTga E207L rev	GGAGTTTTCTAGTTGGGTCTCG
89	PfCRTga E207Q for	CGAGACCCAACAAGAAAACTCC

90	PfCRTga E207Q rev	GGAGTTTTCTTGTTGGGTCTCG
91	PfCRTga E207K for	CGAGACCCAAAAGGAAAACTCC
92	PfCRTga E207K rev	GGAGTTTTCCTTTTGGGTCTCG
93	PfCRTga E207C for	CGAGACCCAATGTGAAAACTCC
94	PfCRTga E207C rev	GGAGTTTTCACATTGGGTCTCG
95	PfCRTga E207F for	CGAGACCCAATTCGAAAACTCC
96	PfCRTga E207F rev	GGAGTTTTCGAATTGGGTCTCG
97	PfCRTga E207H for	CGAGACCCAACACGAAAACTCC
98	PfCRTga E207H rev	GGAGTTTTCGTGTTGGGTCTCG
99	PfCRTga E207N for	CGAGACCCAAAACGAAAACTCC
100	PfCRTga E207N rev	GGAGTTTTCGTTTTGGGTCTCG
101	PfCRTga E207R for	CGAGACCCAACGAGAAAACTCC
102	PfCRTga E207R rev	GGAGTTTTCTCGTTGGGTCTCG
103	PfCRTga E207W for	CGAGACCCAATGGGAAAACTCC
104	PfCRTga E207W rev	GGAGTTTTCCCATTGGGTCTCG
105	PfCRTga E207S for	CCTTCGAGACCCAAAGCGAAAACTCC
106	PfCRTga E207S rev	GGAGTTTTCGCTTTGGGTCTCGAAGG
107	PfCRTga E207T for	CGAGACCCAAACGGAAAACTCC
108	PfCRTga E207T rev	GGAGTTTTCCGTTTGGGTCTCG
109	PfCRTga E207G for	CGAGACCCAAGGAGAAAACTCC
110	PfCRTga E207G rev	GGAGTTTTCTCCTTGGGTCTCG
111	PfCRTga E207I for	CGAGACCCAAATCGAAAACTCC
112	PfCRTga E207I rev	GGAGTTTTCGATTTGGGTCTCG
113	PfCRTga E207P for	CGAGACCCAACCAGAAAACTCC
114	PfCRTga E207P rev	GGAGTTTTCTGGTTGGGTCTCG
115	PfCRTga E207V for	CGAGACCCAAGTGGAAAACTCC
116	PfCRTga E207V rev	GGAGTTTTCCACTTGGGTCTCG
117	PfCRTga R374A for	GTTATTGAACCTGCATTATTGGAC
118	PfCRTga R374A rev	GTCCAATAATGCAGGTTCAATAAC
119	PfCRTga N84A for	GAACCTTGGCCAAGATTGGTAAC
120	PfCRTga N84A rev	GTTACCAATCTTGGCCAAGGTTC
121	PfCRTga K80A for	CCATCTTCGCCGCGAGAACCTTG
122	PfCRTga K80A rev	CAAGGTTCTCGCGGCGAAGATGG

Supplementary Table 7. Composition of systems used in the MD simulations.

		Open-To-Vacuole Protonated E207	Open-To-Vacuole Deprotonated E207	Occluded E207 Protonated	Occluded E207 Deprotonated	Open-To-Cytoplasm Protonated E207	Open-To-Cytoplasm Deprotonated E207
**simulation box dimensions (nm)**		10.9x10.9x10.1	10.9x10.9x9.6	11.0x11.0x10.4	10.9x10.9x10.6	11.1x11.1x9.6	11.0x11.0x9.7
**total number of atoms**		127070	121674	132246	132355	125684	125781
**total number of water molecules**		22701	20903	24474	24511	21784	21817
**number of ions (150 mM of NaCl)**	Na+	55	55	53	53	58	58
	Cl-	69	68	65	64	68	67
**number of lipid molecules and type**	CHL (cholesterol)	138	138	138	138	144	144
	POPC (1-palmitoyl-2-oleoyl-glycero-3-phosphocholine)	184	184	184	184	192	192
	SOPE (1-stearoyl-2-oleoyl-sn-glycero-3-phosphoethanolamine)	138	138	138	138	144	144

Supplementary Methods

Equations describing 16 different models of drug competition

The previously published [4,5] 16 common kinetic models of substrate competition were taken from Segel [6]. The concentration of substrate is denoted as [S1] and the concentration of the inhibiting substrate is denoted as [S2]. K_{S1} and K_{S2} are the dissociation constants for the respective substrate-protein complexes. Full mixed-type inhibition is given by equation (1),

$$v = \frac{V_{max}\frac{[S1]}{K_{S1}}}{1+\frac{[S1]}{K_{S1}}+\frac{[S2]}{K_{S2}}+\frac{[S1][S2]}{\alpha K_{S1}K_{S2}}} \quad (1)$$

Partial mixed-type inhibition is given by equation (2),

$$v = \frac{V_{max}\left(\frac{[S1]}{K_{S1}}+\frac{\beta[S1][S2]}{\alpha K_{S1}K_{S2}}\right)}{1+\frac{[S1]}{K_{S1}}+\frac{[S2]}{K_{S2}}+\frac{[S1][S2]}{\alpha K_{S1}K_{S2}}} \quad (2)$$

Full competitive inhibition is described by equation (3),

$$v = \frac{V_{max}\frac{[S1]}{K_{S1}}}{1+\frac{[S1]}{K_{S1}}+\frac{[S2]}{K_{S2}}} \quad (3)$$

Partial competitive inhibition is described by equation (4),

$$v = \frac{V_{max}\left(\frac{[S1]}{K_{S1}}+\frac{[S1][S2]}{\alpha K_{S1}K_{S2}}\right)}{1+\frac{[S1]}{K_{S1}}+\frac{[S2]}{K_{S2}}+\frac{[S1][S2]}{\alpha K_{S1}K_{S2}}} \quad (4)$$

Full noncompetitive inhibition is given by equation (5),

$$v = \frac{V_{max}\frac{[S1]}{K_{S1}}}{1+\frac{[S1]}{K_{S1}}+\frac{[S2]}{K_{S2}}+\frac{[S1][S2]}{K_{S1}K_{S2}}} \quad (5)$$

Partial noncompetitive inhibition is given by equation (6),

$$v = \frac{V_{max}\left(\frac{[S1]}{K_{S1}}+\frac{\beta[S1][S2]}{K_{S1}K_{S2}}\right)}{1+\frac{[S1]}{K_{S1}}+\frac{[S2]}{K_{S2}}+\frac{[S1][S2]}{K_{S1}K_{S2}}} \quad (6)$$

Full uncompetitive inhibition is described by equation (7),

$$v = \frac{V_{max}\frac{[S1]}{K_{S1}}}{1+\frac{[S1]}{K_{S1}}+\frac{[S1][S2]}{K_{S1}K_{S2}}} \quad (7)$$

Partial uncompetitive inhibition is described by equation (8),

$$\boldsymbol{v} = \frac{\mathbf{V_{max}[S1]}}{\frac{\mathrm{K_{S1}}}{(1+\frac{\beta[\mathrm{S2}]}{\mathrm{K_{S2}}})}+[\mathbf{S1}]\frac{(1+\frac{[\mathrm{S2}]}{\mathrm{K_{S2}}})}{(1+\frac{\beta[\mathrm{S2}]}{\mathrm{K_{S2}}})}} \tag{8}$$

Ligand exclusion is given by equation (9),

$$\boldsymbol{v} = \frac{\mathrm{V_{max}}\left(\frac{[\mathrm{S1}]}{\mathrm{K_{S1}}}+\frac{[\mathrm{S1}]^2}{\mathrm{K_{S1}}^2}\right)}{1+\frac{2[\mathrm{S1}]}{\mathrm{K_{S1}}}+\frac{[\mathrm{S1}]^2}{\mathrm{K_{S1}}^2}+\frac{[\mathrm{S2}]}{\mathrm{K_{S2}}}} \tag{9}$$

Cooperative substrate binding in which the inhibitor mimics the substrate (Cooperative Binding) is given by equation (10),

$$\boldsymbol{v} = \frac{\mathrm{V_{max}}\left(\frac{[\mathrm{S1}]}{\mathrm{K_{S1}}}+\frac{[\mathrm{S1}]^2}{a\mathrm{K_{S1}}^2}+\frac{[\mathrm{S1}][\mathrm{S2}]}{a\mathrm{K_{S1}K_{S2}}}\right)}{1+\frac{2[\mathrm{S1}]}{\mathrm{K_{S1}}}+\frac{[\mathrm{S1}]^2}{a\mathrm{K_{S1}}^2}+\frac{2[\mathrm{S1}][\mathrm{S2}]}{a\mathrm{K_{S1}K_{S2}}}+\frac{2[\mathrm{S2}]}{\mathrm{K_{S2}}}+\frac{[\mathrm{S2}]^2}{a\mathrm{K_{S2}}^2}} \tag{10}$$

Cooperative substrate binding in which the inhibitor does not mimic the substrate (Cooperative Binding 1) is given by equation (11),

$$\boldsymbol{v} = \frac{\mathrm{V_{max}}\left(\frac{[\mathrm{S1}]}{\mathrm{K_{S1}}}+\frac{[\mathrm{S1}]^2}{a\mathrm{K_{S1}}^2}+\frac{[\mathrm{S1}][\mathrm{S2}]}{\mathrm{K_{S1}K_{S2}}}\right)}{1+\frac{2[\mathrm{S1}]}{\mathrm{K_{S1}}}+\frac{[\mathrm{S1}]^2}{a\mathrm{K_{S1}}^2}+\frac{2[\mathrm{S1}][\mathrm{S2}]}{\mathrm{K_{S1}K_{S2}}}+\frac{2[\mathrm{S2}]}{\mathrm{K_{S2}}}+\frac{[\mathrm{S2}]^2}{\mathrm{K_{S2}}^2}} \tag{11}$$

Cooperative inhibitor binding (Cooperative Binding 2) is given by equation (12),

$$\boldsymbol{v} = \frac{\mathrm{V_{max}}\left(\frac{[\mathrm{S1}]}{\mathrm{K_{S1}}}+\frac{[\mathrm{S1}]^2}{\mathrm{K_{S1}}^2}+\frac{[\mathrm{S1}][\mathrm{S2}]}{\mathrm{K_{S1}K_{S2}}}\right)}{1+\frac{2[\mathrm{S1}]}{\mathrm{K_{S1}}}+\frac{[\mathrm{S1}]^2}{\mathrm{K_{S1}}^2}+\frac{2[\mathrm{S1}][\mathrm{S2}]}{\mathrm{K_{S1}K_{S2}}}+\frac{2[\mathrm{S2}]}{\mathrm{K_{S2}}}+\frac{[\mathrm{S2}]^2}{c\mathrm{K_{S2}}^2}} \tag{12}$$

Two-site pure competitive inhibition is given by equation (13),

$$\boldsymbol{v} = \frac{\mathrm{V_{max}}\left(\frac{[\mathrm{S1}]}{\mathrm{K_{S1}}}+\frac{[\mathrm{S1}]^2}{a\mathrm{K_{S1}}^2}+\frac{[\mathrm{S1}][\mathrm{S2}]}{b\mathrm{K_{S1}K_{S2}}}\right)}{1+\frac{2[\mathrm{S1}]}{\mathrm{K_{S1}}}+\frac{[\mathrm{S1}]^2}{a\mathrm{K_{S1}}^2}+\frac{2[\mathrm{S1}][\mathrm{S2}]}{b\mathrm{K_{S1}K_{S2}}}+\frac{2[\mathrm{S2}]}{\mathrm{K_{S2}}}+\frac{[\mathrm{S2}]^2}{c\mathrm{K_{S2}}^2}} \tag{13}$$

For a non-cooperative substrate in the absence of inhibitor in which the substrate reverses the effect of the inhibitor (Substrate non-cooperative), equation (14) is given,

$$\boldsymbol{v} = \frac{\mathrm{V_{max}}\left(\frac{[\mathrm{S1}]}{\mathrm{K_{S1}}}+\frac{[\mathrm{S1}]^2}{\mathrm{K_{S1}}^2}+\frac{[\mathrm{S1}][\mathrm{S2}]}{\alpha\mathrm{K_{S1}K_{S2}}}+\frac{[\mathrm{S1}]^2[\mathrm{S2}]}{\alpha\mathrm{K_{S1}}^2\mathrm{K_{S2}}}\right)}{1+\frac{2[\mathrm{S1}]}{\mathrm{K_{S1}}}+\frac{[\mathrm{S1}]^2}{\mathrm{K_{S1}}^2}+\frac{2[\mathrm{S1}][\mathrm{S2}]}{\alpha\mathrm{K_{S1}K_{S2}}}+\frac{[\mathrm{S2}]}{\mathrm{K_{S2}}}+\frac{[\mathrm{S1}]^2[\mathrm{S2}]}{\alpha\mathrm{K_{S1}}^2\mathrm{K_{S2}}}} \tag{14}$$

For a cooperative substrate in the absence of inhibitor in which the substrate reverses the effect of the inhibitor (Substrate cooperative), equation (15) is given,

$$\boldsymbol{v} = \frac{V_{max}\left(\frac{[S1]}{K_{S1}}+\frac{[S1]^2}{\alpha K_{S1}{}^2}+\frac{[S1][S2]}{\alpha K_{S1}K_{S2}}+\frac{[S1]^2[S2]}{a\alpha K_{S1}{}^2K_{S2}}\right)}{1+\frac{2[S1]}{K_{S1}}+\frac{[S1]^2}{\alpha K_{S1}{}^2}+\frac{2[S1][S2]}{\alpha K_{S1}K_{S2}}+\frac{[S2]}{K_{S2}}+\frac{[S1]^2[S2]}{a\alpha K_{S1}{}^2K_{S2}}} \tag{15}$$

A system in which the inhibitor eliminates substrate cooperativity is given by equation (16),

$$\boldsymbol{v} = \frac{V_{max}\left(\frac{[S1]}{K_{S1}}+\frac{[S1]^2}{\alpha K_{S1}{}^2}+\frac{[S1][S2]}{K_{S1}K_{S2}}+\frac{[S1]^2[S2]}{K_{S1}{}^2K_{S2}}\right)}{1+\frac{2[S1]}{K_{S1}}+\frac{[S1]^2}{\alpha K_{S1}{}^2}+\frac{2[S1][S2]}{K_{S1}K_{S2}}+\frac{[S2]}{K_{S2}}+\frac{[S1]^2[S2]}{K_{S1}{}^2K_{S2}}} \tag{16}$$

Discrimination between drug competition models and statistical analyses of the kinetic data

Analyses of the kinetic data were performed using SigmaPlot version 13.0 and Python. The drug competition models were globally fit to the kinetic data using the least-squares method and ranked according to their corrected Akaike Information Criterion difference (ΔAIC_C) and their Akaike weight using Python. The top-ranked models were compared using an F-test.

The corrected Akaike Information Criterion (AIC_C) of each model was calculated according to equation (17),

$$\mathbf{AIC_C} = \boldsymbol{n} \times \ln\left(\frac{\mathbf{RSS}}{\mathbf{n}}\right) + 2 \times \boldsymbol{K} + \frac{2\times\boldsymbol{K}\times(\boldsymbol{K}+1)}{\boldsymbol{n}-\boldsymbol{K}-1} \tag{17}$$

where RSS is the residual sum of squares (equation (18)),

$$\mathbf{RSS} = \sum_{i=1}^{n}(\mathbf{y_i} - \hat{\mathbf{y}}_i)^2 \tag{18}$$

Here, n is the total number of measurements used to perform the global fit, y_i are the experimentally measured values, $\hat{y}_i$ are the values predicted by the model, and K is the number of parameters in the model. The ΔAIC_C of the ith model (Δ_i) was calculated according to equation (19),

$$\Delta_i = \mathbf{AIC_C^i} - \mathbf{AIC_C^{min}} \tag{19}$$

where the AIC_C^{min} is the smallest AIC_C value of all of the models tested.

For a more detailed evaluation of the plausibility of the models, the Akaike weight (w_i) was calculated according to equation (20),

$$\mathbf{w_i} = \frac{\mathbf{e}^{-\frac{1}{2}\times\Delta_i}}{\sum_{j=1}^{J}\mathbf{e}^{-\frac{1}{2}\times\Delta_j}} \tag{20}$$

where Δ_i is as described above, Δ_j is the AIC_C difference of the *j*th model tested, and J is the total number of models investigated. w_i can range from 0 to 1 and reports the plausibility of the of the *i*th model. The model with the highest Akaike weight is more likely to be correct, and the ratio of the Akaike weight of two models reports how much more likely one model is with respect to the other.

The *F* statistic for the selection between the two best ranking models was calculated according to equation (21), and the corresponding *p* value was obtained using R.

$$\boldsymbol{F} = \frac{\mathbf{RSS_1 - RSS_2}}{\mathbf{RSS_2}} \times \frac{\mathbf{df_2}}{\mathbf{df_1 - df_2}} \tag{21}$$

Here, RSS_1 is the residual sum of squares of the simpler model (the one with less parameters), and RSS_2 is the residual sum of squares of the more complex model (the one with more parameters). df_1 and df_2 are the degrees of freedom of the simpler and more complex model, respectively.

Supplementary References

1. Martin, R. E. *et al.* Chloroquine transport via the malaria parasite's chloroquine resistance transporter. *Science* **325**, 1680-1682 (2009).

2. Bienert, S. *et al.* The SWISS-MODEL Repository-new features and functionality. *Nucleic Acids Res* **45**, D313-d319 (2017).

3. Waterhouse, A. *et al.* SWISS-MODEL: homology modelling of protein structures and complexes. *Nucleic Acids Res* **46**, W296-w303 (2018).

4. Gomez, G. M. *et al.* PfCRT mutations conferring piperaquine resistance in falciparum malaria shape the kinetics of quinoline drug binding and transport. *PLoS Pathog* **19**, e1011436 (2023).

5. Bellanca, S. *et al.* Multiple drugs compete for transport via the Plasmodium falciparum chloroquine resistance transporter at distinct but interdependent sites. *J Biol Chem* **289**, 36336-36351 (2014).

6. Segel, I. H. *Enzyme kinetics: behavior and analysis of rapid equilibrium and steady-state enzyme systems*. Wiley-Interscience (1975).
