# Supplementary material for: pH-dependence of the Plasmodium falciparum chloroquine resistance transporter is linked to the transport cycle
Source: Nat Commun. 2023 Jul 15;14:4234. doi: 10.1038/s41467-023-39969-2 (PMC10349806; doi:10.1038/s41467-023-39969-2)
Supplement: Supplementary file 3 — Reporting Summary [file 41467_2023_39969_MOESM3_ESM.pdf]

nature portfolio

Corresponding author(s): Michael Lanzer

Last updated by author(s): May 12, 2023

Reporting Summary

Nature Portfolio wishes to improve the reproducibility of the work that we publish. This form provides structure for consistency and transparency in reporting. For further information on Nature Portfolio policies, see our Editorial Policies and the Editorial Policy Checklist.

Statistics

For all statistical analyses, confirm that the following items are present in the figure legend, table legend, main text, or Methods section.

n/a	Confirmed	
☐	☒	The exact sample size (n) for each experimental group/condition, given as a discrete number and unit of measurement
☐	☒	A statement on whether measurements were taken from distinct samples or whether the same sample was measured repeatedly
☐	☒	The statistical test(s) used AND whether they are one- or two-sided<br>*Only common tests should be described solely by name; describe more complex techniques in the Methods section.*
☐	☒	A description of all covariates tested
☐	☒	A description of any assumptions or corrections, such as tests of normality and adjustment for multiple comparisons
☐	☒	A full description of the statistical parameters including central tendency (e.g. means) or other basic estimates (e.g. regression coefficient) AND variation (e.g. standard deviation) or associated estimates of uncertainty (e.g. confidence intervals)
☐	☒	For null hypothesis testing, the test statistic (e.g. F, t, r) with confidence intervals, effect sizes, degrees of freedom and P value noted<br>*Give P values as exact values whenever suitable.*
☒	☐	For Bayesian analysis, information on the choice of priors and Markov chain Monte Carlo settings
☒	☐	For hierarchical and complex designs, identification of the appropriate level for tests and full reporting of outcomes
☒	☐	Estimates of effect sizes (e.g. Cohen's d, Pearson's r), indicating how they were calculated

Our web collection on statistics for biologists contains articles on many of the points above.

Software and code

Policy information about availability of computer code

Data collection	ImageJ2 2.3.0/1.53j, Propka 3.5.0, pdb2pqr 2.1.1, Modeller 9.22, Gromacs 2020.1, AMBER FF19SB, Autodock Vina 1.1
Data analysis	Sigma Plot 14.5, Gromacs 2020.1, MDTraj 1.9.8, UCSF chimera 1.17.1, Jupyter Notebook (anaconda3)

For manuscripts utilizing custom algorithms or software that are central to the research but not yet described in published literature, software must be made available to editors and reviewers. We strongly encourage code deposition in a community repository (e.g. GitHub). See the Nature Portfolio guidelines for submitting code & software for further information.

Data

Policy information about availability of data

All manuscripts must include a data availability statement. This statement should provide the following information, where applicable:

- Accession codes, unique identifiers, or web links for publicly available datasets
- A description of any restrictions on data availability
- For clinical datasets or third party data, please ensure that the statement adheres to our policy

The authors declare that the data supporting the findings of this study are available within the article and its supplementary information files, or available from the authors upon request. The experimental protein structures used in this work are available from the protein data bank (PDB entry: 6UKJ (https://doi.org/10.2210/pdb6ukj/pdb), 5Y79 (https://doi.org/10.2210/pdb5y79/pdb)). Source data and code are provided with this paper via the Dryad Digital Repository at https://doi:10.5061/dryad.2z34tmprm. The structural models of PfCRT are available in the Zenodo Digital Repository at https://doi.org/10.5281/zenodo.7828870.

Human research participants

Policy information about studies involving human research participants and Sex and Gender in Research.

Reporting on sex and gender	N/A
Population characteristics	N/A
Recruitment	N/A
Ethics oversight	N/A

Field-specific reporting

Please select the one below that is the best fit for your research. If you are not sure, read the appropriate sections before making your selection.

☒ Life sciences ☐ Behavioural & social sciences ☐ Ecological, evolutionary & environmental sciences

For a reference copy of the document with all sections, see nature.com/documents/nr-reporting-summary-flat.pdf

Life sciences study design

All studies must disclose on these points even when the disclosure is negative.

Sample size	The sample size was calculated using Sigma Plot 13.
Data exclusions	No data were excluded.
Replication	For every system, 3 replicas of MD simulations were run. All other data reported are based on at least three independent biological replicates. Each biological replicate consists of oocytes obtained from a different frog, and at least 10 oocytes analyzed per condition.
Randomization	Samples were not randomly allocated into different groups. This was not possible because we analyzed different mutants.
Blinding	Blinding was not possible. The reason is that we analyzed a large number of mutants.

Behavioural & social sciences study design

All studies must disclose on these points even when the disclosure is negative.

Study description	N/A
Research sample	N/A
Sampling strategy	N/A
Data collection	N/A

	N/A
Timing	N/A
Data exclusions	N/A
Non-participation	N/A
Randomization	N/A

Ecological, evolutionary & environmental sciences study design

All studies must disclose on these points even when the disclosure is negative.

Study description	N/A
Research sample	N/A
Sampling strategy	N/A
Data collection	N/A
Timing and spatial scale	N/A
Data exclusions	N/A
Reproducibility	N/A
Randomization	N/A
Blinding	N/A

Did the study involve field work? ☐ Yes ☒ No

Reporting for specific materials, systems and methods

We require information from authors about some types of materials, experimental systems and methods used in many studies. Here, indicate whether each material, system or method listed is relevant to your study. If you are not sure if a list item applies to your research, read the appropriate section before selecting a response.

Materials & experimental systems

n/a	Involved in the study	
☐	☒	Antibodies
☒	☐	Eukaryotic cell lines
☒	☐	Palaeontology and archaeology
☐	☒	Animals and other organisms
☒	☐	Clinical data
☒	☐	Dual use research of concern

Methods

n/a	Involved in the study	
☒	☐	ChIP-seq
☒	☐	Flow cytometry
☒	☐	MRI-based neuroimaging

Antibodies

Antibodies used	The guinea pig anti-PfCRT antibody was obtained from Eurogentec (Köln, Germany), the anti -guinea pig-POD donkey antibody was obtained from Jacskon ImmunoResearch (706-036-14, Lot 123006, Suffolk, UK). The rabbit anti-PfCRT antibody was a gift from David Fidock. The anti-rabbit goat-POD antibody was obtained from Jackson ImmunoResearch (111-035-045, Lot 130634). The Wheat Germ Agglutinin Alexa Fluor 633 was obtained form Thermo Fisher Scientific (W21404, Lot 1712188, Dreieich, Germany). The anti-mouse-POD goat antibody was obtained from Jackson ImmunoResearch (115-035-166, Lot 131499).
Validation	The antibodies have been validated in Bakouh et al. (2017) and Fidock et al. (2000).

Eukaryotic cell lines

Policy information about cell lines and Sex and Gender in Research

Cell line source(s)	N/A
Authentication	N/A
Mycoplasma contamination	N/A
Commonly misidentified lines (See ICLAC register)	N/A

Palaeontology and Archaeology

Specimen provenance	N/A
Specimen deposition	N/A
Dating methods	N/A

☐ Tick this box to confirm that the raw and calibrated dates are available in the paper or in Supplementary Information.

Ethics oversight	N/A

Note that full information on the approval of the study protocol must also be provided in the manuscript.

Animals and other research organisms

Policy information about studies involving animals; ARRIVE guidelines recommended for reporting animal research, and Sex and Gender in Research

Laboratory animals	Xenopus laevis frogs (> 2 years old)
Wild animals	No wild animals were used in this study
Reporting on sex	Female
Field-collected samples	No field-collected samples were used in this study
Ethics oversight	Ethical approval of the work performed with Xenopus laevis frogs was obtained from the Regierungspräsidium Karlsruhe (Aktenzeichen 35-9185.81/G-31/11 and 35-918581/G-21/23) in accordance with the German "Tierschutzgesetz".

Note that full information on the approval of the study protocol must also be provided in the manuscript.

Clinical data

Policy information about clinical studies
All manuscripts should comply with the ICMJE guidelines for publication of clinical research and a completed CONSORT checklist must be included with all submissions.

Clinical trial registration	N/A

Study protocol	N/A
Data collection	N/A
Outcomes	N/A

Dual use research of concern

Policy information about dual use research of concern

Hazards

Could the accidental, deliberate or reckless misuse of agents or technologies generated in the work, or the application of information presented in the manuscript, pose a threat to:

No	Yes	
☒	☐	Public health
☒	☐	National security
☒	☐	Crops and/or livestock
☒	☐	Ecosystems
☒	☐	Any other significant area

Experiments of concern

Does the work involve any of these experiments of concern:

No	Yes	
☒	☐	Demonstrate how to render a vaccine ineffective
☒	☐	Confer resistance to therapeutically useful antibiotics or antiviral agents
☒	☐	Enhance the virulence of a pathogen or render a nonpathogen virulent
☒	☐	Increase transmissibility of a pathogen
☒	☐	Alter the host range of a pathogen
☒	☐	Enable evasion of diagnostic/detection modalities
☒	☐	Enable the weaponization of a biological agent or toxin
☒	☐	Any other potentially harmful combination of experiments and agents

ChIP-seq

Data deposition

☐ Confirm that both raw and final processed data have been deposited in a public database such as GEO.

☐ Confirm that you have deposited or provided access to graph files (e.g. BED files) for the called peaks.

Data access links<br>*May remain private before publication.*	N/A
Files in database submission	N/A
Genome browser session<br>(e.g. UCSC)	N/A

Methodology

Replicates	N/A
Sequencing depth	N/A
Antibodies	N/A
Peak calling parameters	N/A

Data quality	*N/A*
Software	*N/A*

Flow Cytometry

Plots

Confirm that:

- [ ] The axis labels state the marker and fluorochrome used (e.g. CD4-FITC).
- [ ] The axis scales are clearly visible. Include numbers along axes only for bottom left plot of group (a 'group' is an analysis of identical markers).
- [ ] All plots are contour plots with outliers or pseudocolor plots.
- [ ] A numerical value for number of cells or percentage (with statistics) is provided.

Methodology

Sample preparation	*N/A*
Instrument	*N/A*
Software	*N/A*
Cell population abundance	*N/A*
Gating strategy	*N/A*

- [ ] Tick this box to confirm that a figure exemplifying the gating strategy is provided in the Supplementary Information.

Magnetic resonance imaging

Experimental design

Design type	*N/A*
Design specifications	*N/A*
Behavioral performance measures	*N/A*

Acquisition

Imaging type(s)	*N/A*
Field strength	*N/A*
Sequence & imaging parameters	*N/A*
Area of acquisition	*N/A*

Diffusion MRI ☐ Used ☐ Not used

Preprocessing

Preprocessing software	*N/A*
Normalization	*N/A*
Normalization template	*N/A*

Normalization template | *N/A*

Noise and artifact removal | *N/A*

Volume censoring | *N/A*

Statistical modeling & inference

Model type and settings | *N/A*

Effect(s) tested | *N/A*

Specify type of analysis: ☐ Whole brain ☐ ROI-based ☐ Both

Statistic type for inference (See Eklund et al. 2016) | *N/A*

Correction | *N/A*

Models & analysis

n/a	Involved in the study
☐	☐ Functional and/or effective connectivity
☐	☐ Graph analysis
☐	☐ Multivariate modeling or predictive analysis

Functional and/or effective connectivity | *N/A*

Graph analysis | *N/A*

Multivariate modeling and predictive analysis | *N/A*
